# Epithelial TMPRSS2 impairs glucose homeostasis in obese mice by regulating ghrelin–GLP-1 receptor signaling pathway

**DOI:** 10.1172/jci.insight.203211

**Published:** 2026-03-17

**Authors:** Dilraj Kaur, Sagarika Chakrabarty, Claudius Witzler, Hongjie Wang, Mengwen Wang, Romina Wolz, Petra Wilgenbus, Jens J.N. Posma, Sivaramakrishna Rachakonda, Federico Marini, Valeriya V. Zinina, Sabine Reyda, Rajinikanth Gogiraju, Claudine Graf, Fahumiya Samad, Katrin Schäfer, Christoph Reinhardt, Natalia Soshnikova, Wolfram Ruf, Thati Madhusudhan

**Affiliations:** 1 Center for Thrombosis and Hemostasis (CTH), Medical Centre of the Johannes Gutenberg University, Mainz, Germany.; 2 Department of Immunology and Microbiology, The Scripps Research Institute, La Jolla, California, USA.; 3 Department of Cardiology, Tongji Hospital, Tongji Medical College, Huazhong University of Science and Technology, Wuhan, Hubei, China.; 4 German Breast Group (GBG) Forschungs GmbH, Neu-Isenburg, Germany.; 5 Institute of Medical Biostatistics, Epidemiology and Informatics (IMBEI), Medical Centre of the Johannes Gutenberg University, Mainz, Germany.; 6 Institute of Molecular Medicine, Johannes Gutenberg University Medical Centre, Mainz, Germany.; 7 Department of Cardiology, Medical Centre of the Johannes Gutenberg University, Mainz, Germany.; 8 Department of Cell Biology, San Diego Biomedical Research Institute, San Diego, California, USA.; 9 German Center for Cardiovascular Research, Partner Site Rhein-Main, Mainz, Germany.

**Keywords:** Inflammation, Metabolism, Vascular biology, Coagulation, G protein-coupled receptors, Proteases

## Abstract

Glucagon-like peptide-1 (GLP-1) and glucose-induced insulinotropic polypeptide (GIP) receptor agonists have revolutionized obesity therapy, but causes of obesity-associated dysregulation of endogenous incretin production remain incompletely understood. Here we show that intestinal transmembrane serine protease 2 (TMPRSS2) plays a pivotal role in deregulating anti-diabetic GLP-1 production in obesity. TMPRSS2 is widely coexpressed in intestinal epithelial cells along with its signaling target protease-activated receptor 2 (PAR2). In addition to its role in regulating coagulation protease–mediated adipose tissue inflammation, PAR2 signaling in the gut controls postprandial GIP secretion. TMPRSS2, but not the epithelial cell–expressed proteases FXa or matriptase, activates PAR2 and thereby promotes postprandial GIP release. Accordingly, a PAR2-mutant mouse resistant to TMPRSS2 cleavage is protected from GIP upregulation and diet-induced obesity. In the context of obesity, TMPRSS2 also attenuates bioavailability of the ghrelin pathway and thereby suppresses GLP-1–mediated control of glucose homeostasis. Pharmacological inhibition or genetic deletion of TMPRSS2 restores ghrelin signaling–dependent GLP-1 secretion and GLP-1’s anti-diabetic effects on nutritional glucose homeostasis. Thus, epithelial cell–expressed TMPRSS2, which critically contributes to the lung pathology in SARS-CoV-2 infection, emerges as an intestinal incretin regulator and a potential link between infection and chronic cardiometabolic diseases.

## Abstract 2

GLP-1 based therapeutics revolutionized anti-diabetic and anti-obesity treatments. The current study identifies epithelial TMPRSS2 as a critical regulator of GLP-1 in obesity induced diabetes.

## Graphical abstract

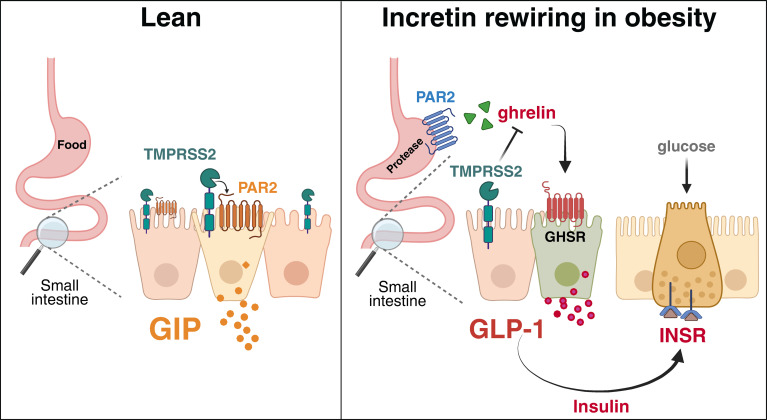

## Introduction

The worldwide dramatic increase in obesity is a major driver of increased all-cause mortality due to cardiometabolic complications and reduced resilience to infectious diseases (1–3). The current and emerging anti-obesity treatments constitute long-lasting analogs of intestinal epithelial cell–expressed incretins that signal through their cognate G protein–coupled glucagon-like peptide-1 (GLP-1) receptor (GLP-1R) and glucose-induced insulinotropic polypeptide (GIP) receptor (GIPR). Postprandially secreted GIP and GLP-1 exert systemic metabolic control by a complex array of signaling mechanisms involving insulinotropic gut-pancreas and satiety-regulating gut-brain axes (4, 5). GLP-1 but not GIP contributes to the defective insulin secretion in type 2 diabetes mellitus (6, 7). GIPR and GLP-1R are widely expressed in various cells of the central nervous system (CNS), adipose tissue, kidney, and cardiovascular and hematopoietic systems, and therefore, effective anti-obesity treatments may have beneficial outcomes beyond reducing obesity (8).

The blood half-life of postprandially generated GIP (4–7 minutes) and GLP-1 (1–2 minutes) is extremely short, and incretins are rapidly degraded by dipeptidyl peptidase 4 (DPP4), which is expressed by various tissue-specific cell types (9). In contrast, the modified pharmacological dual receptor agonists for GIPR and GLP-1R with extended half-life of up to 5 days (120 hours) markedly enhance effects on satiety-regulating pathways in the CNS (5, 10). Experimental and clinical studies with long-acting GLP-1R agonists or unimolecular peptides that simultaneously target GLP-1R and GIPR have shown marked anti-hyperglycemic effects and weight reduction (11–13). In contrast to these pharmacological effects in obesity, animal studies largely depict obesity-promoting effects of GIP and attribute the weight-regulating effects of GIP to CNS-GIPR signaling (14). Intriguingly, pharmacological GIPR agonism combined with GLP-1R agonism also involves CNS-GIPR signaling in the control of satiety pathways (15, 16), suggesting rewiring of incretin signaling in obesity.

Postprandial gut hormone secretion by enteroendocrine cells involves a wide variety of signaling pathways, including ion channels, nutrient transporters, and GPCRs (17, 18). Food uptake particularly exposes small-intestinal epithelial cells to activated or released digestive and microbiota-associated proteases. The GPCR protease-activated receptor 2 (PAR2) is expressed on the luminal and basolateral side of epithelial cells and is a promiscuous sensor for a wide range of endogenous and bacterial proteases, which are known to regulate epithelial permeability and intestinal inflammation (19, 20). Disease-specific alterations of bacterial proteolytic activity in celiac and Crohn’s disease enhance inflammation dependently on PAR2 cleavage (21, 22). Additionally, PAR2-dependent signaling has been implicated in promoting weight gain and metabolic inflammation in mouse models of diet-induced obesity (DIO) (23), but the specific proteases by which PAR2 signaling exerts metabolic inflammation and DIO remain unclear (24).

Protease-specific PAR2 activation drives both protective and inflammatory pathways in a context-dependent manner (25). PAR2 can heterodimerize with PAR1, or PAR3, and other transmembrane receptors, exerting distinct G protein– or PAR2/β-arrestin–mediated intracellular responses (26–28). This complex receptor crosstalk of PAR2, however, can be disrupted in global and cell type–specific knockouts of this GPCR (29). To avoid this pitfall, we here use PAR2-mutant mice (PAR2-G37I and PAR2-R38E) that retain activation-independent signaling receptor platforms involving PAR2, while abolishing cleavage sensitivity to specific proteases (21, 22, 30, 31). In combination with serine protease–knockout mice, we delineate the roles of intestinal PAR2-activating proteases in postprandial incretin secretion and alterations in DIO. Our study reveals epithelial cell–expressed intestinal transmembrane serine protease 2 (TMPRSS2), which plays a pivotal role in SARS-CoV-2 cell entry and infection (32), as a key regulator of postprandial gut hormone balance and obesity-induced diabetes. Delineation of the obesity-specific roles of TMPRSS2 offers potential therapeutic opportunities for rebalancing host metabolic derangements during obesity-related complications including viral infections.

## Results

### PAR2 and activating proteases are coexpressed in enterocytes.

**Figure 1:**
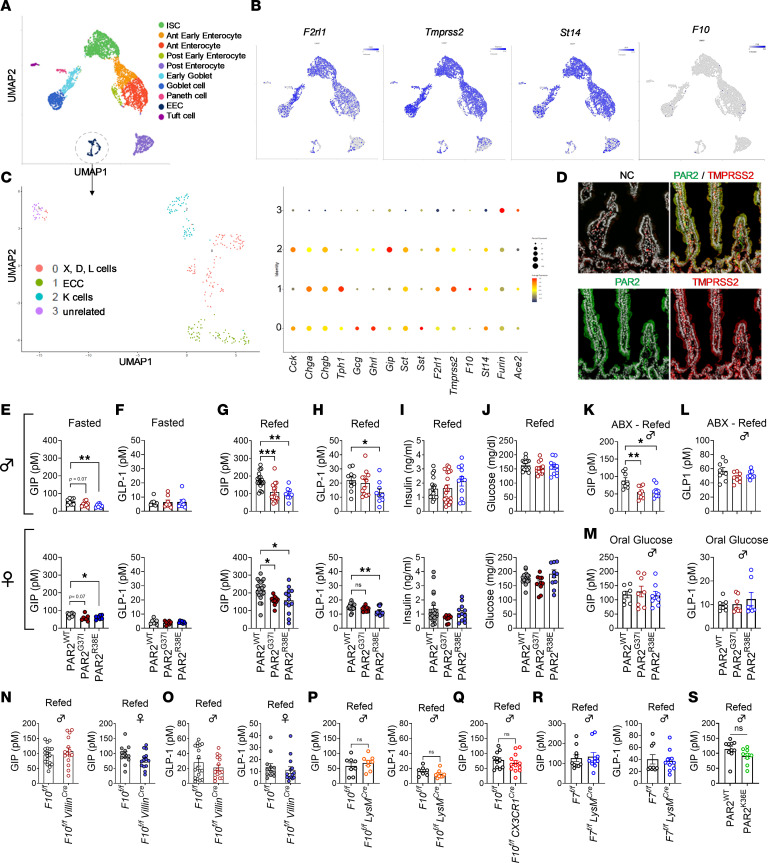
Protease-specific PAR2 signaling regulates postprandial incretin secretion. (**A**) Uniform manifold approximation and projection (UMAP) plot showing clustering of intestinal epithelial cell types isolated from the crypts of WT mice and analyzed by single-cell RNA sequencing. (**B**) Cell type–specific expression of *F2rl1* and PAR2-activating proteases *St14*, *Tmprss2*, and *F10*. (**C**) Subclusters of enteroendocrine cells expressing gut hormones and proteases. (**D**) Immunofluorescence images showing colocalization of PAR2 and TMPRSS2 in the mouse small intestine. Original magnification ×40. (**E** and **F**) Plasma concentrations of GIP (**E**) and GLP-1 (**F**) in fasted PAR2-mutant mice. (**G**–**J**) Refeeding experiments with PAR2-mutant mice. Plasma GIP (**G**), GLP-1 (**H**), insulin (**I**), and blood glucose (**J**) levels in non-obese mice fasted overnight and refed for 1 hour. (**K** and **L**) Postprandial GIP (**K**) and GLP-1 (**L**) levels in mice treated for 2 weeks with broad-spectrum antibiotics. (**M**) Plasma concentrations of GIP and GLP-1 in mice fasted overnight followed by the administration of oral glucose (2 g/kg body weight) for 15 minutes. (**N**–**R**) Refeeding experiments in coagulation factor–deficient mice. (**N** and **O**) Postprandial GIP (**N**) and GLP-1 (**O**) levels in refed mice with intestinal epithelial cell–specific deletion of *F10*. (**P**) GIP and GLP-1 levels in refed mice with myeloid cell–specific deletion of *F10*. (**Q**) Postprandial GIP in macrophage *F10*-deficient mice. (**R**) GIP and GLP-1 levels in refed mice with myeloid cell–specific deletion of *F7*. (**S**) Postprandial GIP levels in refed PAR2^K36E^ mice. Data represent mean ± SEM. (**E**–**S**) One-way ANOVA. *P < 0.05, **P < 0.01, ***P < 0.001. ISC, intestinal stem cells; ECC, enteroendocrine cells; NC, negative control.

PAR2 is activated by several soluble trypsin-like proteases and multiple transmembrane-anchored serine proteases (TMPRSSs) (33–35), but their roles in PAR2-mediated metabolic control are incompletely understood. To identify potential PAR2-activating proteases expressed in the gut, we performed single-cell RNA sequencing of intestinal crypt cells and identified subpopulations by distinct marker transcripts (Figure 1A and Supplemental Figure 1A; supplemental material available online with this article; https://doi.org/10.1172/jci.insight.203211DS1). Transcripts for the PAR2-activating proteases matriptase (*St14*) and TMPRSS2 (*Tmprss2*) were coexpressed by various enterocyte populations in lean mice. The majority of the enterocytes expressed both *Tmprss2* and *St14*, and nearly 45% coexpressed *F2rl1* encoding PAR2 (Figure 1, A and B). Additionally, within the enteroendocrine cells both *F2rl1* and *Tmprss2* were expressed in *Gip*-producing cells as well as in tryptophan hydroxylase 1–positive (*Tph1*-positive) enterochromaffin cells (Figure 1C). The PAR2-activating coagulation protease FXa (*F10*), known to be expressed in various extrahepatic cell types, had low mRNA expression in the intestinal crypt cells at our sequencing depth, but a survey of recently published single-cell gene expression data showed that *F10* was expressed in proximal enterocytes, whereas *Tmprss2* and *F2rl1* predominantly colocalized in the early enterocytes (36). Immunohistochemical analysis of WT mouse small intestine sections provided corroborating evidence for our single-cell expression data and showed that PAR2 and TMPRSS2 colocalized in crypt regions and throughout the villi of the small intestine (Figure 1D).

### PAR2-G37I mutation attenuates postprandial GIP in lean mice, independent of sex.

Given the colocalization of PAR2 within the enteroendocrine populations, we hypothesized that protease-specific PAR2 signaling in the gut might modulate postprandial incretin secretion. To this end, we used mice with a point mutation in PAR2 at Gly^37^ to Ile (PAR2-G37I mice) that abrogated proteolytic activation by matriptase and coagulation FXa, but maintained sensitivity to other proteases, including trypsin (30, 31). Additionally, in mice with a point mutation of PAR2 at Arg^38^ to Glu (PAR2-R38E mice), PAR2 was rendered insensitive to activation at the canonical cleavage site by bacterial and endogenous trypsin-like proteases (21, 22, 30, 31).

To determine the homeostatic roles of protease-specific PAR2 signaling for gut hormone secretion, we measured baseline plasma concentrations of the incretins GIP and GLP-1 in lean mice under fasted conditions. When compared with PAR2-WT mice, fasted PAR2-G37I mice showed a trend toward reduced GIP secretion, whereas both male and female fasted PAR2-R38E mice showed significantly reduced GIP levels (Figure 1E) without alterations in GLP-1 levels (Figure 1F). To analyze postprandial incretin secretion, we fasted PAR2-mutant and WT mice overnight followed by refeeding of normal chow diet (NCD) for 1 hour, a reliable time point as determined by a pilot experiment in WT mice on both C57BL/6N and C57BL/6J backgrounds (Supplemental Figure 1B). Refed lean male and female PAR2-G37I mice displayed significantly reduced postprandial GIP levels (Figure 1G), whereas postprandial GLP-1 levels were not significantly different from those in PAR2-WT mice (Figure 1H). In contrast, lean male and female PAR2-R38E mice showed a significant reduction not only in postprandial GIP, but also in GLP-1 levels in the plasma (Figure 1, G and H), suggesting that additional proteases directly or indirectly participated in GLP-1 regulation specifically during food intake.

The altered homeostatic incretin levels in PAR2-G37I or PAR2-R38E versus WT mice were not caused by apparent changes in metabolism, as no significant differences in food and fluid intake, energy expenditure, respiratory exchange ratio, or locomotor activity were observed in indirect calorimetry (Supplemental Figure 2, A and B). Altered incretin levels were also not associated with significant differences in postprandial insulin and glucose levels measured at the same time (Figure 1, I and J). We reason that the residual GIP and GLP-1 levels are sufficient to maintain glucose homeostasis in a lean mouse.

The composition of gut microbiota influences the postprandial levels of gut hormones in healthy and diseased animals. Depletion of gut microbiota had no effect on the suppressed postprandial GIP levels in PAR2-G37I or PAR2-R38E mice when compared with PAR2-WT mice (Figure 1K). However, antibiotic treatment increased postprandial GLP-1 levels relative to colonized mice and abolished the differences seen in PAR2-R38E mice relative to PAR2-WT and PAR2-G37I mice (Figure 1L). The efficient depletion of gut microbiota after antibiotic treatment was confirmed by detection of bacterial 16S DNA (Supplemental Figure 1C). These data suggested that gut microbiota–mediated PAR2 activation, which is attenuated in PAR2-R38E mice (21, 22), contributed to the alteration in postprandial homeostatic GLP-1 levels.

Apart from food intake, oral glucose administration induces GIP and GLP-1 secretion dependently on the intestine-specific glucose transporters sodium glucose cotransporter-1 (SGLT1) and glucose transporter 2 (GLUT2) (37, 38). Unlike food intake, oral administration of a glucose bolus revealed no significant differences in GIP and GLP-1 levels in PAR2 mutants versus WT mice (Figure 1M), suggesting that food uptake induced specific proteolytic alterations that especially contributed to GIP release. Furthermore, gene expression analysis of intestinal epithelial cells (IECs) showed no significant differences in mRNA levels of *Gip* and *Glp1* and the potential upstream transcription factor regulatory factor X6 (*Rfx6*) in duodenum, jejunum, ileum, and colon, while expression of pancreatic and duodenal homeobox 1 (*Pdx1*) was higher in jejunum of PAR2-R38E mice when compared with PAR2-G37I and WT mice (Supplemental Figure 2C). While the known technical difficulties of isolating high-quality intestinal RNA after refeeding prevented us from excluding a transcriptional regulation of *Gip*, GIP was clearly regulated at the protein level in fasted and refed mice by PAR2 signaling (39).

Postprandial gut hormones are regulated by DPP4 expressed by gut epithelial, endothelial, and immune cells (9), and systemic DPP4 has been implicated in FXa/PAR2-mediated adipose tissue inflammation (40). We therefore characterized the composition of immune cell types as possible modulators of GIP secretion by single-cell RNA sequencing of CD45^+^ cells isolated from the intestinal lamina propria. PAR2-G37I mice showed no apparent differences in the composition of immune cell types compared with PAR2-WT mice (Supplemental Figure 3, A–C). In addition, *DPP4* was similarly expressed in monocytes (cluster 6) and macrophages (cluster 4) in PAR2-G37I versus PAR2-WT mice (Supplemental Figure 3D). We also directly measured postprandial DPP4 activity in peripheral blood mononuclear cells, small intestine (SI), and plasma (Supplemental Figure 3E). DPP4 activity did not differ between PAR2-G37I and PAR2-WT mice, indicating that differences in GIP levels observed in PAR2-G37I mice were not due to altered DPP4 expression in immune cells or DPP4 activity.

### Epithelial and myeloid cell–derived FXa is dispensable for homeostatic GIP regulation.

We next determined the role of intestinal FXa for postprandial GIP regulation by deleting FX with the Villin^Cre^ driver in IECs (Supplemental Figure 3F). Compared with WT littermate *F10^f/f^* mice, male and female *F10^f/f^ Villin^Cre^* mice showed unaltered postprandial GIP and GLP-1 levels (Figure 1, N and O), as well as IEC transcript levels of *Gip* and *Glp1* and the upstream transcription factors *Rfx6* and *Pdx1* (Supplemental Figure 3G). Based on prior data implicating macrophage FXa as a PAR2 activator in obesity (40), we next addressed the role of myeloid cell–derived FXa for the regulation of GIP. Lean *F10^f/f^ LysM^Cre^* mice also showed no difference in postprandial GIP or GLP-1 levels relative to WT littermate *F10^f/f^* mice (Figure 1P). Expression of CX3CR1 marks tissue-resident macrophages, including in the intestine (41), but macrophage deletion of FX in *F10^f/f^ CX3CR1^Cre^* mice also did not attenuate postprandial GIP levels (Figure 1Q). Experiments with mice that lack the FX activator FVII in myeloid cells (28) also did not show an effect on postprandial GIP and GLP-1 levels (Figure 1R). These data indicated that FX derived from IECs or macrophages had no role in the homeostatic regulation of postprandial GIP.

Given the expression of *St14* (matriptase) in the intestinal crypt cells, we next determined the role of matriptase-PAR2 signaling for postprandial GIP regulation by using PAR2-K36E mutant mice. This mutant is resistant to cleavage by matriptase but sensitive to PAR2 activation by FXa-like proteases (31). Unlike FXa-resistant PAR2-G37I mice, lean PAR2-K36E mice showed no difference in postprandial GIP levels, excluding a role for the matriptase-PAR2 axis in GIP regulation (Figure 1S).

### TMPRSS2 is the PAR2-activating protease in GIP regulation.

**Figure 2:**
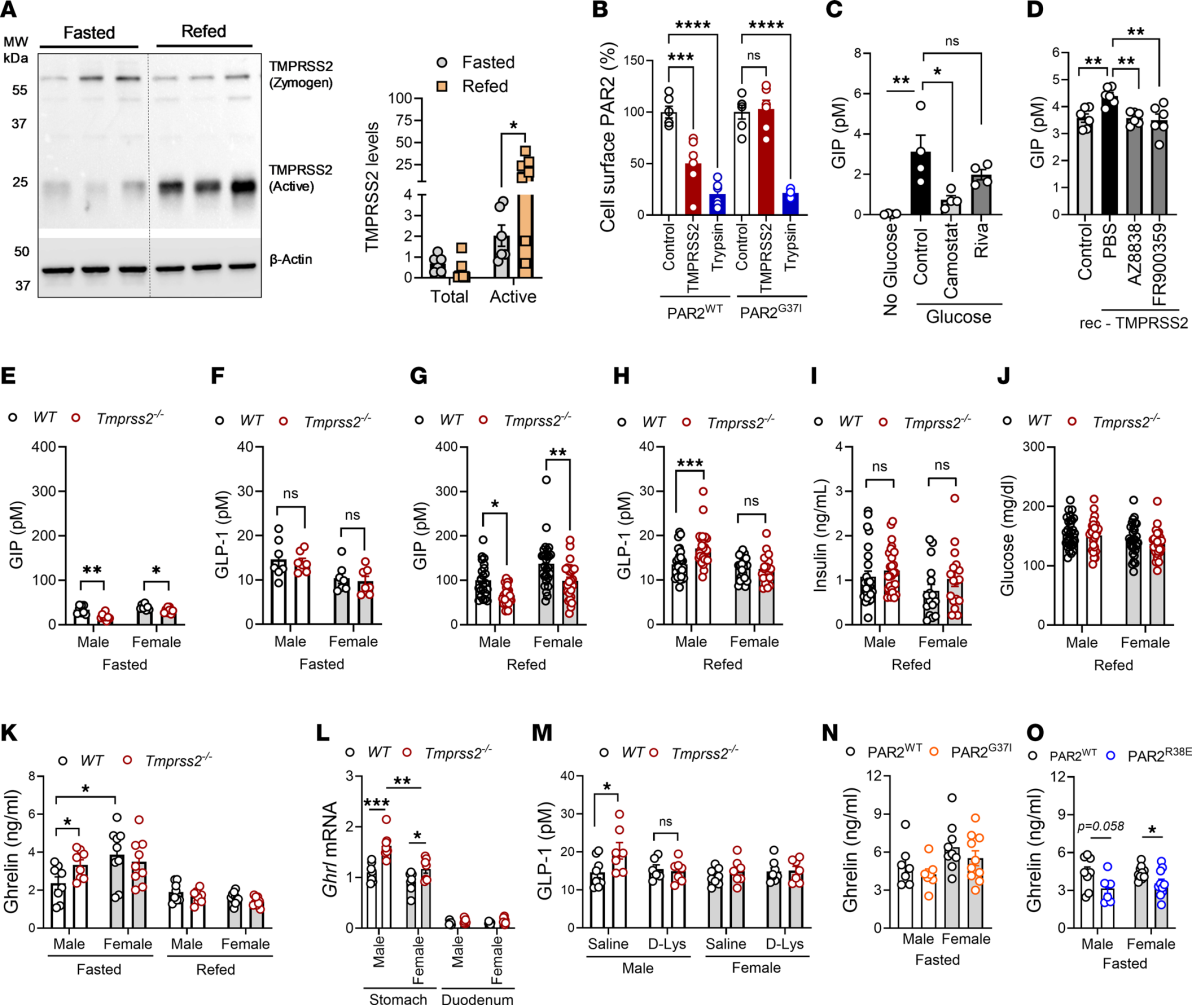
TMPRSS2 is the PAR2-activating protease regulating GIP. (**A**) Quantification of TMPRSS2 protein levels in the intestinal epithelial cells isolated from lean WT fasted and refed mice. Mean ± SEM; *n* ≥ 6 mice per group. (**B**) Quantification of PAR2 cleavage by TMPRSS2 and trypsin in a cell surface ELISA for FLAG-tagged PAR2. Residual PAR2 expression was determined after 60 minutes of incubation. (**C** and **D**) GIP levels measured in culture supernatants of GLUTag cells. (**E** and **F**) Plasma concentrations of GIP (**E**) and GLP-1 (**F**) in fasted *Tmprss2^–/–^* and WT mice. (**G**–**J**) Postprandial GIP (**G**), GLP-1 (**H**), insulin (**I**), and blood glucose (**J**) levels in mice fasted overnight and refed for 1 hour. (**K**) Plasma concentrations of ghrelin in overnight-fasted and 1 hour–refed lean male and female *Tmprss2^–/–^* and WT mice. (**L**) *Ghrl* mRNA levels in stomach and intestinal epithelial cells isolated from duodenum of *Tmprss2^–/–^* and WT mice. (**M**) Plasma concentrations of GLP-1 in fasted and 1-hour–refed mice. The ghrelin receptor antagonist d-Lys3 was administered intraperitoneally in a subset of mice before refeeding. (**N**–**O**) Plasma concentrations of ghrelin in fasted lean male and female PAR2-mutant and corresponding WT mice. Data represent mean ± SEM. (**B**) One-way ANOVA; (**A** and **C**–**O**) 2-tailed *t* test. *P < 0.05, **P < 0.01, ***P < 0.001, ****P < 0.0001.

While matriptase has been implicated in the regulation of IEC functions (20, 34), homeostatic roles of TMPRSS2 in the intestine are unknown. Membrane-bound TMPRSS2 zymogen undergoes autocleavage activation at the Arg^255^-Ile^256^ peptide bond, and the matured enzyme proteolytically activates membrane proteins of the host and invading pathogens (42). We next analyzed whether active TMPRSS2 is an integral component of nutrition-induced proteolytic cascades in the IECs that modulates postprandial GIP secretion. When compared with fasted WT mice, refeeding robustly induced activation of TMPRSS2 in the IECs isolated from mouse proximal small intestine (Figure 2A). We measured PAR2 cleavage by purified active TMPRSS2 in a cellular assay with amino-terminally FLAG-tagged PAR2 constructs transfected into CHO cells (43). PAR2-WT and PAR2-G37I were efficiently cleaved by trypsin. Indeed, TMPRSS2 also caused a loss of the surface FLAG tag in cells transfected with PAR2-WT, but, remarkably, not with PAR2-G37I (Figure 2B). These results demonstrated that TMPRSS2 cleaves PAR2 and that the PAR2-G37I mutant is resistant to proteolytic activation by TMPRSS2.

We next performed a GIP secretion assay in an L cell model (GLUTag cells) (44), which, like other cancer-derived cell lines, also produces GIP (45). Treatment with the serine protease inhibitor camostat, which inhibits, among other proteases, TMPRSS2, reduced glucose-stimulated GIP secretion in GLUTag cells (34), whereas treatment with the FXa-specific inhibitor rivaroxaban showed no significant difference (Figure 2C). Congruently, treatment with recombinant TMPRSS2 induced GIP secretion, which was abrogated by pretreatment with either the PAR2 antagonist AZ8838 or the Gα_q_ inhibitor FR900359 (Figure 2D). We next analyzed *Tmprss2^–/–^* mice for IEC transcript levels of *Gip* and *Glp1*, which showed no differences between male and female *Tmprss2^–/–^* and WT mice at baseline (Supplemental Figure 4, A and B). Thus, TMPRSS2 does not regulate *Gip* transcription in vivo.

However, *Tmprss2^–/–^* mice displayed reduced baseline plasma concentrations of GIP under fasted conditions (Figure 2E), but fasted GLP-1 levels were not significantly different from those in strain-matched WT mice (Figure 2F). In line with the PAR2-G37I phenotype, lean male and female *Tmprss2^–/–^* mice showed reduced postprandial GIP secretion when compared with WT mice (Figure 2G). However, unlike PAR2-G37I mice, male but not female *Tmprss2^–/–^* mice also showed enhanced postprandial plasma GLP-1 levels (Figure 2H). Despite differences in postprandial GLP-1 levels, both male and female *Tmprss2^–/–^* mice showed a trend toward higher postprandial blood insulin levels (Figure 2I) without apparent changes in postprandial blood glucose levels (Figure 2J). In response to food intake, GLP-1 is produced by L cells predominantly present in the distal part of the small intestine (46). Quantification of IECs double-positive for epithelial cell adhesion molecule (EpCAM) and GLP-1 by flow cytometry showed no differences between *Tmprss2^–/–^* and WT mice, indicating regulation of GLP-1 secretion (Supplemental Figure 4, C and D). Metabolic profiling provided no evidence for changes in locomotor activity, lower food intake or water intake, or changes in energy expenditure or respiratory exchange ratio (Supplemental Figure 4, E–I) between lean WT and *Tmprss2^–/–^* mice. In addition, glucose tolerance test (GTT) or oral glucose challenge showed no apparent differences between lean WT and *Tmprss2^–/–^* mice (Supplemental Figure 4, J and K). Thus, the similar postprandial reduction of GIP levels in male and female *Tmprss2^–/–^* and PAR2-G37I mice indicates that TMPRSS2-dependent PAR2 signaling regulates GIP secretion.

### Ghrelin is required for increased GLP-1 production in male Tmprss2^–/–^ mice.

Increased postprandial GLP-1 levels observed predominantly in male *Tmprss2^–/–^* mice indicated an additional sex-specific mechanism unrelated to the activation of PAR2 by TMPRSS2. GLP-1 is in part regulated by the hunger hormone ghrelin (47). Ghrelin levels are induced during fasting but rapidly decline after feeding. Recent studies have shown that ghrelin receptor antagonism inhibits GLP-1 production induced by oral glucose challenge (47, 48). Ghrelin levels in fasted male *Tmprss2^–/–^* mice were elevated relative to male WT mice and reached the significantly higher ghrelin levels found in females (Figure 2K). As expected, refeeding induced a rapid decline in ghrelin to similar levels in female and male WT and *Tmprss2^–/–^* mice (Figure 2K). We found that both male and female *Tmprss2^–/–^* mice had increased ghrelin-encoding *Ghrl* mRNA levels in the stomach compared with WT mice (Figure 2L), indicating that post-transcriptional mechanisms primarily determine the significantly higher ghrelin levels in *Tmprss2^–/–^* mice.

To investigate the role of ghrelin for the regulation of postprandial GLP-1 by TMPRSS2, we inhibited ghrelin signaling with the ghrelin receptor antagonist [d-Lys3]-GHRP-6 (47) before refeeding. Compared with vehicle-treated mice, pretreatment with d-Lys3 abolished the increase in postprandial GLP-1 in male *Tmprss2^–/–^* mice but had no effect on GLP-1 levels in male WT mice (Figure 2M). In addition, no significant differences in postprandial GLP-1 levels were observed between saline-treated and d-Lys3–treated female *Tmprss2^–/–^* and WT mice (Figure 2M). Also, TMPRSS2-insensitive PAR2-G37I mice showed no increases in plasma ghrelin levels and thus did not phenocopy *Tmprss2^–/–^* mice (Figure 2N).

Surprisingly, PAR2-R38E mice showed reduced ghrelin levels in both sexes, indicating that an alternative protease, such as trypsin expressed by epithelial cells of the stomach (49), directly regulated ghrelin expression through PAR2 signaling (Figure 2O). Notably, the lower ghrelin levels in PAR2-R38E mice were also associated with reduced postprandial GLP-1 levels (Figure 1H), emphasizing the role of ghrelin for postprandial GLP-1 secretion. The increased ghrelin levels in *Tmprss2^–/–^* mice could therefore not simply be explained by a loss of PAR2 activation but might involve a negative regulatory role of TMPRSS2 on PAR2 signaling by an alternative protease controlling ghrelin expression. Thus, TMPRSS2 repression of ghrelin levels represents an important mechanism controlling GLP-1 production predominantly in male mice in the absence of obesity.

### PAR2-G37I mice are protected from DIO independent of sex.

**Figure 3:**
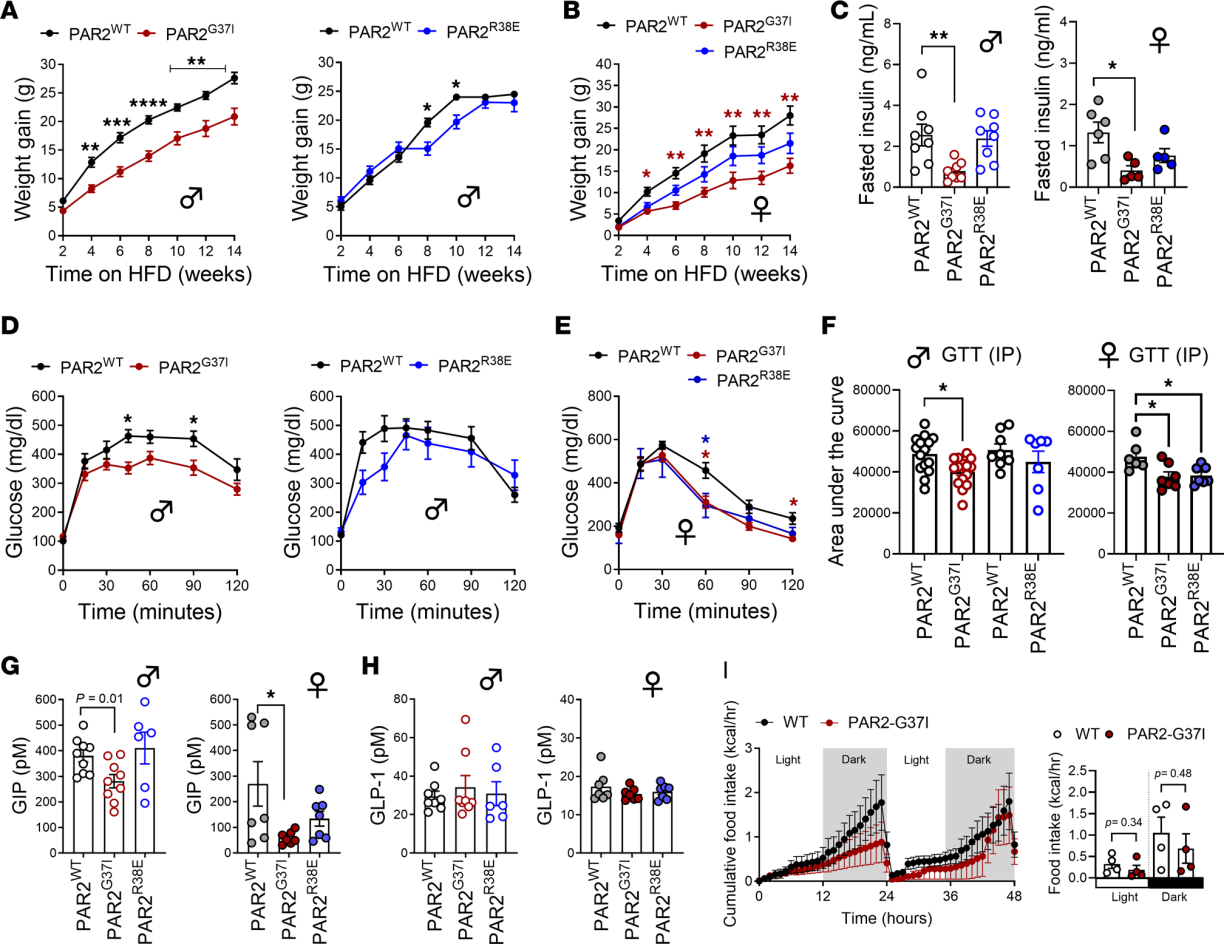
Protease-selective PAR2 activation promotes DIO. (**A** and **B**) Weight gain of PAR2-G37I, PAR2-R38E, and WT male (*n* = 19/16/13) (**A**) and female (*n* = 7/7/7) (**B**) mice on HFD. (**C**) Fasted plasma insulin levels in mice on a HFD for 14 weeks. (**D**–**F**) Glucose tolerance test (GTT) following intraperitoneal glucose injection in male PAR2-G37I and WT (*n* = 15/15) and PAR2-R38E and WT (*n* = 7/8) (**D**) or female PAR2-G37I, PAR2-R38E, and WT (*n* = 7/7/6) (**E**) and quantification of area under the curve for both male and female PAR2-G37I and PAR2-R38E versus WT mice (**F**). (**G** and **H**) Postprandial GIP (**G**) and GLP-1 (**H**) levels in PAR2-mutant mice on HFD for 10 weeks. Mice were fasted overnight and refed HFD for 1 hour before blood collection to determine postprandial hormone concentrations in plasma. (**I**) Food intake (kcal/h) in HFD-fed PAR2-G37I and WT mice. Data represent mean ± SEM. (**A**, **B**, **D**, and **E**) Longitudinal data were analyzed by 2-way ANOVA with time and genotype as covariables, Šidák’s multiple-comparison test. All others, 1-way ANOVA. *P < 0.05, **P < 0.01, ***P < 0.001.

Given the crucial role of protease-specific PAR2 signaling in regulation of postprandial GIP, we next conducted DIO studies in PAR2-G37I and PAR2-R38E mutant mice. PAR2-G37I and PAR2-R38E mice on a normal chow diet (NCD) showed no differences in body weight, fasting glucose, and insulin levels compared with WT mice (Supplemental Figure 5, A–C). However, PAR2-G37I male mice on a high-fat diet (HFD) for 14 weeks showed significantly reduced weight gain compared with WT mice (Figure 3A). In contrast, weight gain differences between male PAR2-R38E and WT mice were less pronounced (Figure 3A). DIO studies in female PAR2 mutant mice gave concordant results, and female PAR2-R38E mice showed only a tendency toward reduced weight gain (Figure 3B). Thus, the phenotypes of PAR2 mutant mice with differential protease sensitivity indicate a critical role for selective protease activation of PAR2 in regulating metabolism in DIO.

The attenuated weight gain in both male and female PAR2-G37I mice, but not PAR2-R38E mice, was associated with significantly reduced hyperinsulinemia in fasted mice (Figure 3C). Accordingly, male PAR2-G37I mice showed improved glucose tolerance in a GTT, whereas male PAR2-R38E mice showed a partial, insignificant reduction in glucose levels (Figure 3, D and F). Significantly improved GTT was also observed in the female PAR2-G37I and PAR2-R38E mice (Figure 3, E and F), and the improved glucose control in female PAR2-R38E mice was associated with a trend toward lower fasting insulin levels (Figure 3C).

In line with only marginally attenuated DIO of male and female PAR2-R38E mice, postprandial gut hormone levels were not different from those in obese PAR2-WT mice (Figure 3, G and H). As in lean PAR2-G37I mice postprandial GIP levels (Figure 3G) but not GLP-1 levels (Figure 3H) were significantly reduced in obese PAR2-G37I mice of both sexes. These data are in agreement with previous reports that GIP antagonism or genetic deletion of GIPR ameliorates DIO (50). Attenuation of DIO in male PAR2-G37I mice was associated with no significant differences in food intake, water intake, locomotor activity, energy expenditure, and respiratory exchange ratio (Figure 3I and Supplemental Figure 5, D–G). These data in both male and female obese mice uncovered a critical role of protease-selective PAR2 activation in the regulation of DIO and postprandial GIP release in obesity.

### Macrophage-autonomous FXa-PAR2 signaling suppresses adipose tissue inflammation.

Obesity development in both rodents and humans is associated with accumulation of adipose tissue macrophages (ATMs), which propagate chronic local and systemic inflammatory responses and thereby contribute to insulin resistance (51). To delineate the protease-specific roles of FXa-PAR2 signaling in adipose tissue inflammation, we isolated the adipose tissue stromal vascular fraction (SVF) from obese PAR2-G37I and WT mice for immunophenotyping. DIO triggers the accumulation of ATMs into crown-like structures around dead adipocytes, and these ATMs typically express CD11c and other markers characteristic of classically activated macrophages (52). Attenuation of DIO in PAR2-G37I mice was associated with significantly reduced numbers of total CD11b^+^ and CD11b^+^CD11c^+^ ATMs within the SVF with a reciprocal increase in CD11b^–^CD11c^–^ and unchanged CD11b^–^CD11c^+^ cells (Supplemental Figure 5H). The CD11b^+^CD11c^+^F4/80^+^ population showed reduced expression of CD206, indicating, surprisingly, a more proinflammatory phenotype in PAR2-G37I mice. No significant differences in CD8^+^ and CD4^+^ T cell and B220^+^ B cell numbers were observed between obese PAR2-G37I and WT mice (Supplemental Figure 5J). However, preadipocyte cells expressing Sca1^+^CD29^+^CD34^+^CD24^+^ showed a trend toward lower numbers in the SVF, which was associated with reduced muscle fat content in PAR2-G37I compared with WT mice (Supplemental Figure 5, K and L).

**Figure 4:**
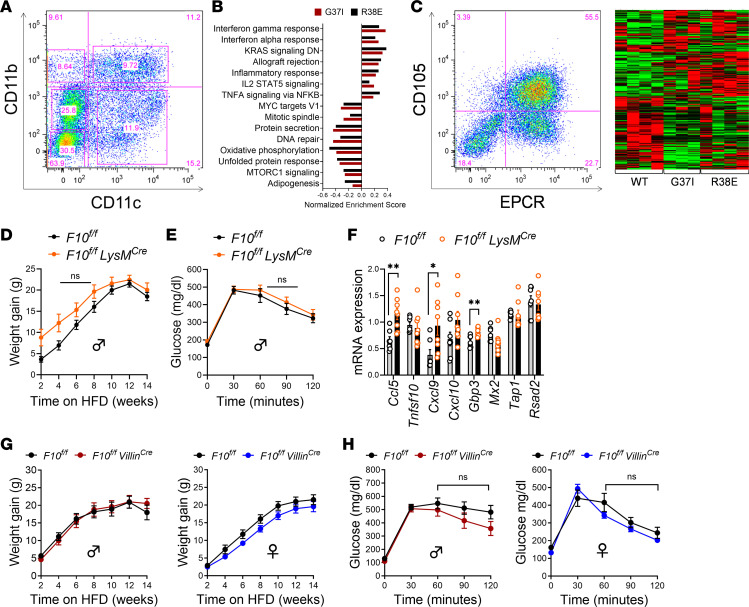
FXa-PAR2 signaling promotes adipose tissue inflammation but not obesity. (**A**) Flow cytometry analysis of ATMs isolated from mice 16 weeks on HFD. (**B**) Normalized enrichment score (NES) of differentially regulated pathways in FACS-isolated CD11b^+^CD11c^+^ ATMs. (**C**) Representative histogram showing flow cytometry analysis of sorted CD105^+^EPCR^+^ endothelial cells (left) and heatmap showing differentially regulated genes in endothelial cells (right) from PAR2-G37I, PAR2-R38E, and WT mice (*n* = 3/4/4). (**D**) Weight gain of *F10^f/f^ LysM^Cre^* and littermate control *F10^f/f^* mice on a HFD for 14 weeks (*n* = 8/10). (**E**) Glucose tolerance test after 14 weeks of HFD in *F10^f/f^ LysM^Cre^* and littermate control *F10^f/f^* mice (*n* = 13/14). (**F**) Gene expression analysis by quantitative PCR of bead-selected CD11c^+^ ATMs isolated from mice on HFD for 14 weeks; *F10^f/f^ LysM^Cre^* (*n* = 8) and littermate control *F10^f/f^* mice (*n* = 7). (**G**) Weight gain of male (*n* = 14/17) and female (*n* = 15/11) *F10^f/f^ Villin^Cre^* and littermate *F10^f/f^* control mice on a HFD for 14 weeks. (**H**) Intraperitoneal glucose tolerance test following intraperitoneal glucose in male (*n* = 8/9) and female (*n* = 9/6) *F10^f/f^ Villin^Cre^* and littermate *F10^f/f^* control mice on a HFD for 14 weeks. Data represent mean ± SEM. (**D**, **E**, **G**, and **H**) Two-way ANOVA with time and genotype as covariables, Šidák’s multiple-comparison test; (**F**) 2-tailed *t* test. *P < 0.05, **P < 0.01.

To better characterize the CD11b^+^CD11c^+^ ATMs, we sorted these cells for transcriptome profiling (Figure 4A). Gene set enrichment analysis of ATMs from obese PAR2-G37I mice indicated a pronounced proinflammatory phenotype, characterized by upregulated signatures associated with interferon response, KRAS signaling, allograft rejection, and IL-2–STAT5 signaling. In contrast, pathways typically linked to cellular protection, including MYC targets V1, protein secretion, DNA repair, oxidative phosphorylation, the unfolded protein response, MTORC1 signaling, and adipogenesis, were markedly downregulated (Figure 4B and Supplemental Figure 6A). Remarkably, this proinflammatory ATM polarization phenotype was also observed in PAR2-R38E when compared with PAR2-WT mice (Figure 4B and Supplemental Figure 6B). Next, we also analyzed the expression profile of SVF microvascular endothelial cells from PAR2-G37I and PAR2-R38E mice, which showed a concordant regulation of transcripts related to inflammatory activation in comparison with obese PAR2-WT mice (Figure 4C). Both mutants likewise exhibited a similar downregulation of genes (Supplemental Figure 7A). Collectively, these data indicated that the more pronounced weight loss phenotype of PAR2-G37I versus PAR2-R38E mice is not explained by differences in ATM or adipose tissue endothelial cell reprogramming.

Interrogation of the ATM data revealed specific proinflammatory transcripts, including the interferon response genes *Gbp3* and *Mx2*, CXCL chemokine family members (*Cxcl19*, *Cxcl11*, *Cxcl10*), and C-C motif chemokine ligand 5 (*Ccl5*), that were commonly upregulated in PAR2 mutants when compared with PAR2-WT mice (Supplemental Figure 7B). Thus, the loss of proteolytic activation of PAR2 incompletely phenocopied the previously demonstrated antiinflammatory phenotype of obese PAR2-knockout mice (23, 40). These divergent results may be explained by impaired Toll-like receptor 4 (TLR4) (25, 53) signaling due to disruption of PAR2 heterodimer formation in the complete PAR2 knockout, whereas selective abrogation of PAR2 cleavage is known to preserve proinflammatory responses but abolishes interferon responses downstream of TLR4 signaling in innate immune cells (54, 55).

PAR2 can also dampen inflammatory responses (56), and myeloid cell–synthesized FXa plays a pivotal role in PAR2-mediated immune suppression in the tumor microenvironment (30). The observed upregulation of inflammatory transcripts in ATMs of PAR2-G37I mice therefore indicated a loss of macrophage-specific FXa-PAR2 signaling. When compared with WT littermate *F10^f/f^* mice, *F10^f/f^ LysM^Cre^* mice showed an initial tendency of increased weight gain (Figure 4D) and were not protected from DIO-induced glucose intolerance (Figure 4E). In line with ATMs from obese PAR2-mutant mice, the gene expression analysis of CD11c-selected ATMs showed increased expression of markers of inflammation (*Ccl5*, *Cxcl9*, *Cxcl10*, and *Gbp3*) in obese *F10^f/f^ LysM^Cre^* compared with the littermate *F10^f/f^* mice (Figure 4F). Thus, the loss of FXa-dependent PAR2 signaling in PAR2-G37I mice and genetic deletion of FX in myeloid cells promoted a concordant macrophage inflammatory phenotype.

However, only PAR2-G37I but not *F10^f/f^ LysM^Cre^* mice were protected from obesity, supporting an obesity-promoting role of protease-selective PAR2 activation that is unrelated to ATMs. We therefore determined the role of intestinal FX for the regulation of obesity and conducted DIO studies in *F10^f/f^ Villin^Cre^* mice. As in *F10^f/f^ LysM^Cre^* mice, DIO studies in both male and female mice revealed no significant differences in HFD-induced weight gain (Figure 4G) or GTT (Figure 4H) between littermate *F10^f/f^* mice and *F10^f/f^ Villin^Cre^* mice. These data demonstrated that IEC-derived FX is also dispensable for the development of DIO.

### TMPRSS2-regulated GLP-1 secretion dominates postprandial glucose control in obesity.

**Figure 5:**
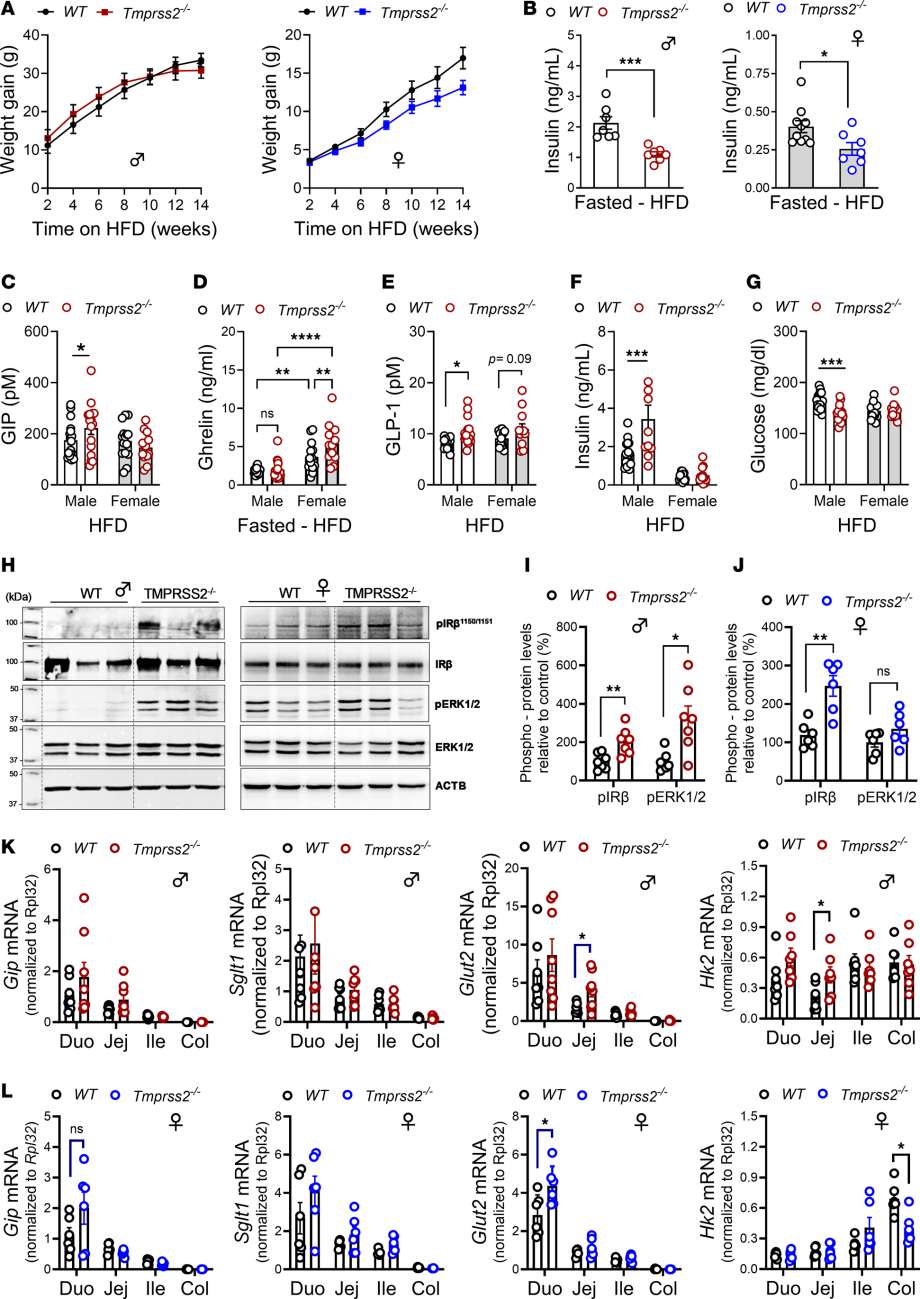
TMPRSS2 ablation improves glucose metabolism in DIO. (**A** and **B**) Weight gain (male, *n* = 22/24; female, *n* = 13/15) (**A**) and fasted plasma insulin concentrations (**B**) in male and female mice on a HFD for 14 weeks. (**C**) Postprandial GIP levels in mice fasted overnight and refed HFD for 1 hour at week 10. (**D**) Plasma concentrations of ghrelin in fasted male and female *Tmprss2^–/–^* and WT mice on a HFD for 14 weeks. (**E**–**G**) Postprandial GLP-1 (**E**), insulin (**F**), and glucose (**G**) levels in mice fasted overnight and refed HFD for 1 hour after HFD for 10 weeks. (**H**–**J**) Representative immunoblots (**H**) show levels of phosphorylated insulin receptor β (IRβ) and downstream p-ERK1/2 in jejunal IECs of obese male (**I**) and female (**J**) HFD-fed WT and *Tmprss2^–/–^* mice. (**K** and **L**) Gene expression analysis of IECs isolated from obese male (**K**) and female (**L**) *Tmprss2^–/–^* and WT mice. Data represent mean ± SEM. (**D**) Two-way ANOVA; (**B**, **C**, **E**–**G**, and **I**–**L**) 2-tailed *t* test; (**A**) 2-way ANOVA with time and genotype as covariables, Šidák’s multiple-comparison test. *P < 0.05, **P < 0.01, ***P < 0.001, ****P < 0.0001.

We next evaluated the impact of the alternative intestinal PAR2 activator TMPRSS2 on DIO. In contrast to the marked protection from weight gain observed in PAR2-G37I mice, male *Tmprss2^–/–^* mice initially gained more weight than WT mice and later exhibited a trend toward reduced weight gain, which was also observed in female *Tmprss2^–/–^* mice throughout the HFD feeding (Figure 5A). These changes were associated with no significant differences in metabolic parameters between *Tmprss2^–/–^* and WT mice (Supplemental Figure 8, A–F). Despite a modest reduction in obesity, male and female *Tmprss2^–/–^* mice showed significantly lower fasting insulin levels (Figure 5B). Postprandial GIP production was elevated in male but unchanged in female *Tmprss2^–/–^* mice compared with WT (Figure 5C), possibly explaining the initial tendency toward increased weight gain in male *Tmprss2^–/–^* versus WT mice.

As observed under non-obese conditions, female mice exhibited higher fasting ghrelin levels than males when fed a HFD (Figure 5D). However, unlike in lean *Tmprss2^–/–^* mice, fasting ghrelin levels were not significantly different in HFD males and, rather, elevated in female *Tmprss2^–/–^* compared with WT mice (Figure 5D). In line with lean male mice, postprandial GLP-1 production was significantly increased in obese male *Tmprss2^–/–^* mice and showed a trend toward higher levels in female *Tmprss2^–/–^* mice compared with WT mice (Figure 5E). These changes were accompanied by increased postprandial insulin secretion (Figure 5F) and reduced glucose levels (Figure 5G) especially in male *Tmprss2^–/–^* compared with WT mice. Collectively, these data indicate that GLP-1 is non-redundantly regulated in both lean and obese *Tmprss2^–/–^* mice, which is in contrast to the primary effect of GIP suppression observed in PAR2-G37I mice.

We next investigated whether altered insulin production in *Tmprss2^–/–^* mice led to enhanced local insulin signaling in the intestine. IECs isolated from the jejunum of HFD *Tmprss2^–/–^* mice showed increased phosphorylation of insulin receptor β (p-IRβ) and extracellular signal–regulated kinase (p-ERK1/2) compared with WT mice (Figure 5, H–J). Congruently, gene expression analysis of IECs relative to respective WT controls showed elevated glucose transporter 2 (*Glut2*) and glycolytic enzyme hexokinase 2 (*Hk2*) expression in the jejunum of the male *Tmprss2^–/–^* mice (Figure 5K), whereas female *Tmprss2^–/–^* mice showed increased *Glut2* expression in the duodenum (Figure 5L). There was also a trend toward higher *Gip* expression in both sexes, which aligned with a recent report showing that insulin receptor signaling in IECs specifically regulates postprandial GIP but not GLP-1 (57). These findings suggest that the obesity-induced rewiring of intestinal incretin signaling in *Tmprss2^–/–^* mice indicates an important feedback loop, where obesity-associated hyperinsulinemia sustains postprandial GIP secretion.

### Ghrelin–GLP-1 signaling improves glucose homeostasis in TMPRSS2 deficiency.

**Figure 6:**
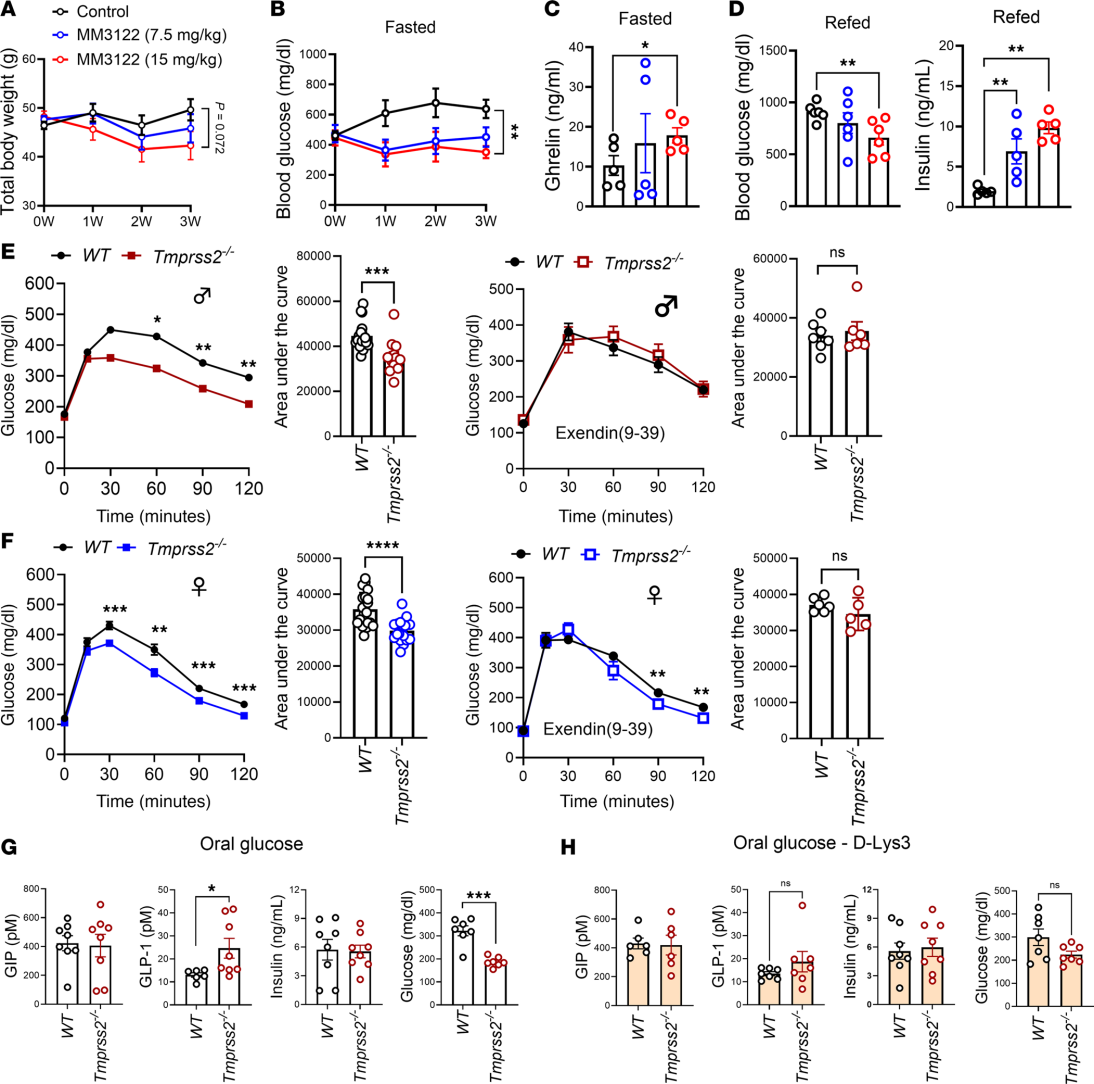
TMPRSS2 suppresses endogenous GLP-1–mediated glucose homeostasis in DIO. (**A**) Body weights (*n* = 6/6/6) of male db/db mice treated with either the TMPRSS2 inhibitor MM3122 or vehicle (saline) as control. (**B** and **C**) Blood glucose levels (**B**) and plasma concentrations of ghrelin (**C**) measured in overnight-fasted db/db mice. (**D**) Blood glucose levels and plasma concentrations of insulin in db/db mice fasted overnight and refed for 1 hour. (**E** and **F**) Oral glucose challenge of fasted obese male (**E**) and female (**F**) *Tmprss2^–/–^* and WT mice with and without pretreatment with the GLP-1R antagonist exendin(9–39) for 15 minutes. Bar graphs show quantification of area under the curve for both males and females. (**G**) Plasma concentrations of GIP, GLP-1, insulin, and glucose 15 minutes after oral glucose administration to fasted mice on HFD for 14 weeks. (**H**) GIP, GLP-1, insulin, and glucose levels in mice treated with the ghrelin receptor antagonist d-Lys3 before glucose administration. Data represent mean ± SEM. (**A**–**D**, **G**, and **H**) Two-tailed t test; (**E** and **F**) 2-way ANOVA with time and genotype as covariables, Šidák’s multiple-comparison test; (**E** and **F**, bar graphs) 2-tailed *t* test. *P < 0.05, **P < 0.01, ***P < 0.001, ****P < 0.0001.

To ascertain the obesity-specific role of TMPRSS2 in regulating postprandial glucose homeostasis, we employed an independent preclinical model of obesity (db/db mice) and performed a pharmacological intervention with a TMPRSS2 inhibitor. Mice homozygous for the spontaneous (*Lepr^db^*) mutation develop severe obesity starting at 3–4 weeks of age, accompanied by overnutrition-related complications including postprandial hyperglycemia, and eventually pancreatic β cell atrophy and hypoinsulinemia. We treated 10-week-old, obese db/db mice by daily oral administration of the TMPRSS2 inhibitor MM3122 (58). After 3 weeks of treatment, mice exhibited a modest though statistically insignificant reduction in total body weight compared with control mice treated with vehicle (saline) (Figure 6A). Treatment also improved fasting glucose levels (Figure 6B). Like *Tmprss2^–/–^* mice, MM3122-treated db/db mice showed elevated fasting ghrelin levels (Figure 6C) along with postprandial reductions in blood glucose and increases in insulin levels (Figure 6D). These findings support the role of TMPRSS2 as a regulator of ghrelin and provide initial evidence for its potential as a therapeutic target in this obesity-associated impairment of glucose homeostasis.

While the experiments above addressed postprandial glucose homeostasis in the context of food intake, we next investigated the mechanism following acute oral glucose administration. Oral glucose administration rapidly induces GLP-1 (47), and obese *Tmprss2^–/–^* mice showed a marked improvement in oral glucose metabolism in comparison with WT mice (Figure 6E). Importantly, blockade of GLP-1R with the antagonist exendin(9–39) (59, 60) prior to the oral glucose challenge completely abolished the improved oral glucose metabolism in male *Tmprss2^–/–^* and largely abolished the improvement in female *Tmprss2^–/–^* compared with WT mice (Figure 6, E and F). Measurements of incretin levels showed that oral glucose administration rapidly induced higher GLP-1 levels in *Tmprss2^–/–^* mice compared with WT mice (Figure 6G). Higher GLP-1 levels were associated with reduced blood glucose levels, whereas GIP and insulin levels remained unchanged in obese *Tmprss2^–/–^* versus WT mice (Figure 6G). Consistent with the role of ghrelin signaling in regulating postprandial GLP-1 in lean mice, administration of the ghrelin receptor antagonist d-Lys3 abolished both the increased GLP-1 levels and the reduction in blood glucose levels observed in obese male *Tmprss2^–/–^* mice following oral glucose challenge (Figure 6H). These data demonstrate that male *Tmprss2^–/–^* mice retain remarkable sensitivity to glucoregulatory ghrelin-dependent GLP-1 production during obesity. Taken together, our data uncovered a critical role of TMPRSS2 as an endogenous regulator of postprandial incretin levels and suggest that targeting TMPRSS2 may represent a potential interventional strategy to improve glucose homeostasis in existing obesity.

## Discussion

The present study identifies an important function of the intestinal protease TMPRSS2 in fine-tuning postprandial metabolic responses. Our results delineate that TMPRSS2-PAR2 signaling regulates postprandial GIP release in lean mice, but that this signaling axis is overlaid by additional functions of TMPRSS2 in the context of hypercaloric weight gain and the associated dysregulation of glucose homeostasis and insulin resistance. TMPRSS2 signaling through PAR2 has been demonstrated in previous studies in cancer biology (61), but our study uncovers an unexpected role for this epithelial cell–expressed protease in activating PAR2 in the regulation of metabolism.

Based on the initial finding that FXa-resistant PAR2-G37I mice are protected from obesity, we have systematically interrogated PAR2-activating roles of FX expressed by innate immune cells or IECs. These experiments essentially exclude a metabolic role for extrahepatic FX synthesis in the activation of PAR2 outside of the vascular system but delineate an important regulatory role of FXa-PAR2 signaling in controlling ATM activation and adipose tissue inflammation. Deletion of FX in myeloid cells amplifies ATM inflammatory responses, which are typically induced by obesity. These effects are similarly observed in FXa-resistant PAR2-G37I mice, suggesting cell-autonomous, autocrine FXa-PAR2 signaling. Despite signs of increased adipose tissue inflammation, only a moderate and insignificant increase in weight gain is observed during the early stages of DIO in *F10^f/f^ LysM^Cre^* mice. The altered ATM phenotype therefore cannot explain the marked attenuation in weight gain seen in FXa cleavage–resistant PAR2-G37I mice as opposed to completely cleavage-resistant PAR2-R38E mice. In addition, deletions of FX from myeloid cells or IECs do not phenocopy the marked reduction of postprandial GIP release observed in PAR2-G37I mice.

However, deletion of TMPRSS2 attenuates postprandial GIP in lean mice similarly to the PAR2-G37I mutation. Corroborating these data, the coexpression of *PAR2* and *Tmprss2* is predominantly observed in various proximal enterocyte populations within the intestinal crypts and enteroendocrine cells. Intriguingly, *Tmprss2* deletion induces increased postprandial GLP-1 levels dependent on ghrelin signaling in lean male mice. Given that PAR2-G37I mutation has no impact on fasted ghrelin or postprandial GLP-1 levels, the mechanism by which TMPRSS2 regulates GLP-1 is likely independent of canonical PAR2 cleavage by TMPRSS2. Unlike the PAR2-G37I mutant, PAR2-R38E mice are insensitive to a wide range of proteases that may have homeostatic functions (21, 22). Importantly, PAR2-R38E mice show a marked reduction in fasting ghrelin levels as well as postprandial GLP-1 levels. This effect and the failure to maintain suppressed GIP levels, as seen in PAR2-G37I mice, may compromise obesity protection in PAR2-R38E mice. The surprising preservation of ghrelin/GLP-1–mediated homeostatic glucose regulation in obese *Tmprss2^–/–^* mice raises the possibility that TMPRSS2 is embedded in protease regulatory networks that especially contribute to the adverse metabolic complications of obesity.

The epithelium of the gastrointestinal tract is exposed to a variety of trypsin-like proteases that can activate PAR2. While this study identifies an important function of epithelial cell–expressed protease beyond controlling digestive processes, we did not analyze all relevant proteases involved in PAR2-mediated homeostatic incretin regulation. Whereas the TMPRSS2-PAR2 pathway is a predominant homeostatic driver of GIP in physiological conditions, in obesity TMPRSS2 deletion fails to reduce GIP and, presumably, GIP-dependent weight gain. We reason that in chronic obesity, sustained GLP-1 activation in *Tmprss2^–/–^* mice improves insulin sensitivity and glucose metabolism but, at the same time, paradoxically regulates insulin-induced GIP secretion independent of PAR2 signaling, thus superseding TMPRSS2 deficiency. In contrast, PAR2-G37I mice display unaltered postprandial GLP-1 and ghrelin levels. Consequently, GIP levels in DIO remain low in PAR2-G37I mice, which show markedly attenuated weight gain. Collectively, our findings underline the emergence of TMPRSS2 as a specific regulator of not only GLP-1 but also ghrelin, both of which play critical roles in metabolic health and disease.

Epidemiological and clinical data from the recent SARS-CoV-2 pandemic indicate that the highest mortality rate is observed in patients suffering from metabolic diseases including obesity and diabetes (62, 63). Interventions targeting metabolic disease may reduce the risk of mortality in COVID-19 (64). Whereas severe COVID-19 disease is clinically dominated by respiratory failure due to thrombo-inflammation and microvascular endothelial dysfunction, SARS-CoV-2 can infect the gastrointestinal tract (65), and persistent infection of the IECs has been linked to the lingering symptoms in long COVID (66, 67). Our results identify a common pathway that is at the interface of propagating both enteric viral infections and disruption of glucose homeostasis. Targeting the TMPRSS2-PAR2 pathway may provide therapeutic opportunities for cardiometabolic disorders and infectious diseases linked to metabolic dysregulation.

## Methods

Further information can be found in Supplemental Methods.

### Sex as a biological variable.

The study involved animal experiments conducted in both male and female mice cohoused in the same animal rooms. Analysis of healthy mice and mice with diet-induced obesity was conducted simultaneously in age-matched mice, preferably littermates, to avoid confounding results.

### Animal experiments.

PAR2-G37I [*B6(Cg)-F2rl1^tm2.1Wmrf^/Tarc*] and PAR2-R38E [*B6(Cg)-F2rl1^tm1.1Wmrf^/Tarc*] mutant mice were generated by homologous recombination of sequence-confirmed mutated *F2rl1* exon 2 with flanking introns 1 and 2 into pBS-FRT-Neo-FRT in C2 C57BL/6N embryonic stem cells at the Scripps Transgenic Core Facility (30). After germline transmission and excision of the resistance cassette by crossing with a flippase deleter strain, the mutants were bred to homozygosity, and the entire coding sequence of the targeted exon was confirmed by DNA sequencing. PAR2^flox^ [*B6(Cg)-F2rl1^tm3.1Wmrf^/Tarc*] were used as wild-type controls (PAR2-WT). The PAR2-K36E [*B6N(Cg)-F2rl1^em1Wmrf^/Tarc*] was generated by pronuclear injection and CRISPR-mediated targeting to change the F2rl1 coding sequence from 5′-AGTAAAGGAAGAAGT-3′ (S K G R S) to 5′-AGTgAAGGAcGAtcg-3′ (S E G R S), which also introduced the diagnostic silent PvuI restriction site. Founders were bred in C57BL/6N mice to homozygosity and the sequence confirmed by genomic sequencing (31). Mutants were crossed with C57BL/6N for the generation of littermate progeny of mutant and WT mice. *F10^f/f^* [*B6(Cg)-F10^tm1c(EUCOMM)/Hmgu^/Tarc*] were crossed with *LysM^Cre^* [*Lyz2^tm1(cre)Ifo^*], *CX3CR1^Cre^* [*Cx3cr1^tm1.1(cre)jung^*], or *Villin^Cre^* [*Tg^(Vil1-cre)1000Gum^*] mice on a C57BL/6N background (Charles River) to generate mice with myeloid cells and intestinal cell–specific deletion mutants. Cre-negative littermates were used as controls (30). The TMPRSS2 knockout (*B6.129-Tmprss2^tm1Tsyk^/JHzif/Tarc*), referred to as *Tmprss2^–/–^*, was generated by Peter S. Nelson (68) and provided after backcrossing with C57BL/6J by Klaus Schughart, Helmholtz Zentrum, Braunschweig, Germany.

Male db/db (*BKS-Lepr^em2Cd479^/Gpt*) mice (GemPharmatech) were housed in a temperature- and humidity-controlled animal facility under a 12-hour light/12-hour dark cycle with free access to standard chow and water. Ten-week-old mice were randomly divided into 3 groups: vehicle control and TMPRSS2 inhibitor (MM3122) 7.5 mg/kg and 15 mg/kg. MM3122 was dissolved in saline and administered once daily by oral gavage at approximately 10 am for 3 consecutive weeks. During the treatment period, body weight and fasted blood glucose concentrations were measured weekly between 8 and 9 am. At the end of the third week, blood was collected from the facial vein of fasted mice to determine blood glucose levels and plasma concentrations of ghrelin. Mice were then allowed to refeed for 1 hour, and blood samples were collected again from the facial vein to determine blood glucose and plasma insulin concentrations.

### Cell lines and isolation of primary IECs.

All the cell lines were grown at 37°C and 5% CO_2_ in a humidified atmosphere. Primary intestinal epithelial cells (IECs) were isolated as described earlier (36). We dissected the whole intestine devoid of stomach and then divided it into the specific anatomical small intestine regions of proximal duodenum, jejunum, and ileum as well as colon. These intestinal sections were flushed with ice-cold phosphate-buffered saline (PBS) to remove fecal matter and placed in 1- to 2-cm pieces into 15 mL sterile tubes with 5 mL dissociation buffer (PBS, 10 mM EDTA) for incubation at 37°C in a shaker at 250 rpm for 20 minutes. The supernatants containing epithelial cells were collected and washed once with sterile cold PBS for RNA isolation, immunoblotting, or flow cytometry. For immunoblotting, IEC pellets were resuspended in RIPA buffer containing protease inhibitor cocktail. After 30 minutes of incubation, lysates were centrifuged at 12,000*g*, and the supernatants were collected for protein estimation and immunoblotting. For flow cytometry, cells from the third villus fraction and crypts were fixed with 4% paraformaldehyde for 15 minutes at room temperature, washed twice with PBS, and permeabilized with saponin-based buffer. Fixed cells were stained with an antibody targeting GLP-1 for 2 hours at room temperature, followed by staining with donkey anti-rabbit Alexa Fluor 488 Plus antibody and APC-conjugated anti–mouse CD326 (EpCAM) antibody for 1 hour at room temperature. Single cells were gated based on DAPI dye intensity. The acquisition was performed using a BD FACSAria III SORP cell sorter (100 μm nozzle) and analyzed using FlowJo software v10.10.0 (FlowJo LLC).

### DIO studies and glucose tolerance tests.

Adult (8-week-old), sex-matched mice were fed rodent diet with 60 kcal% fat from Research Diets (D12492) for up to 14 weeks. Normal chow diet (NCD) was obtained from Ssniff Diets. For intraperitoneal or oral glucose tolerance tests (GTTs), mice were fasted overnight for 12–14 hours followed by the administration of glucose (2 g/kg body weight) intraperitoneally or through oral gavage. After administration of glucose, blood glucose levels were measured from minor tail pricks at desired time intervals (0, 15, 30, 60, 90, and 120 minutes) using a glucometer (CONTOUR NEXT, Ascensia Diabetes Care). In a subset of mice, GLP-1R antagonist (exendin-3, Tocris) was administered intraperitoneally before oral glucose challenge.

### Postprandial hormone analysis.

Adult (10- to 12-week-old), sex-matched mice obtained from the same breeding colony and maintained on an NCD were used for the postprandial hormone analysis, whereas obese mice were analyzed 10 weeks after HFD feeding. Mice were fasted overnight for 12–14 hours followed by refeeding with the respective diets. One hour after refeeding, blood samples were collected via cheek bleeds or cardiac puncture, and plasma was isolated and snap-frozen for subsequent hormone analysis by ELISA. Mouse total GIP ELISA kit (81257) and GLP-1 ELISA kit (81508) were obtained from Crystal Chem. Mouse Ghrelin ELISA kit (EZRGT-91K) was from Merck, and Mouse Insulin ELISA kit (80-INSMS-E01) was from ALPCO. For glucose-stimulated gut hormone secretion in mice, glucose (2 g/kg body weight) was administered by oral gavage, and blood samples were collected via cheek bleeds 15 minutes later to determine plasma concentrations of gut hormones and insulin and blood glucose levels. In a subset of mice, the ghrelin antagonist [d-Lys^3^]-GHRP-6 (G4535, Sigma-Aldrich) was administered intraperitoneally 15 minutes before oral glucose administration.

### Antibiotic treatment.

To deplete gut microbiota, animals received a cocktail of 100 μg/mL neomycin, 50 μg/mL streptomycin, 100 μg/mL ampicillin, 50 μg/mL vancomycin, 100 μg/mL metronidazole, 1 mg/mL bacitracin, 125 μg/mL ciprofloxacin, and 100 μg/mL ceftazidime (Sigma-Aldrich) in the drinking water (69). Antibiotic-containing drinking water was prepared freshly every 2 days. Two weeks after initiation of antibiotic treatment, postprandial blood samples were collected to determine plasma gut hormone concentrations by ELISA.

### Mouse metabolic phenotyping.

The metabolic phenotyping of mice was performed in single-housed mice fed with either NCD or HFD (Promethion, Sable Systems). The mice were adapted for at least 5 days to the single-housing environment before the recording of the metabolic data. Metabolic measurement in the Promethion Core Line (Sable Systems) provides a controlled environment that allows measurement of a series of metabolic and behavioral parameters, including respiratory exchange ratio, energy expenditure, oxygen consumption (V_O2_), carbon dioxide production (V_CO2_), locomotor activity, and food and water consumption. Each metabolic cage is equipped with various sensors, including independent mass analyzer, flow controller, and pumps, that allow for the continuous measurement of the animal’s metabolic activity. According to the manufacturer’s recommendation, mice were initially weighed and transferred individually into the 8 metabolic cages with free access to food and water. Temperature of the program was kept constant at 22°C, and lights were set to turn on at 6 am and off at 6 pm to match the circadian cycle of the mouse. After the program was started, metabolic and behavioral parameters were recorded every 5 minutes into the system over a specific period of time. At the end, the mice were again weighed, and later metabolic parameters were adjusted to the mouse body mass recorded at the start of the experiment using CalR software (calorimetry R language–based software; https://calrapp.org).

### Immunohistochemistry.

Adult mouse small intestines were fixed for 20 minutes in 1% formaldehyde in PBS at room temperature, incubated overnight in 30% sucrose, embedded in OCT compound, and kept at –80°C. Immunohistochemical analyses were performed on 15 μm cryosections. Sections were blocked with 5% donkey serum in 0.1% NP-40/PBS at room temperature for 1 hour. Primary antibodies targeting TMPRSS2 (1:100; Sc-515727, Santa Cruz Biotechnology) and PAR2 (1:250; made in-house) were incubated overnight at 4°C, followed by staining with donkey anti-rabbit Alexa Fluor 488 Plus antibody (1:1,000; Thermo Fisher Scientific) and donkey anti-mouse Alexa Fluor 594 antibody (1:1,000; Thermo Fisher Scientific) for 1 hour at room temperature. Counterstaining of nuclei was performed with DAPI (1:1,000; Sigma-Aldrich). Sections were embedded in Vectashield (Vector Laboratories). Images were acquired with a Leica SP8 confocal microscope.

### PAR2 cleavage assay.

PAR2 cleavage was quantified on Chinese hamster ovary (CHO)–K1 cells stably transfected with human TF and endothelial protein C receptor (EPCR) and transiently transfected with FLAG-tagged PAR2-WT or PAR2-G37I mutants, as described previously (43). Cell surface PAR2 was detected by anti-FLAG antibody (Sigma-Aldrich) in a cell-based ELISA assay that measured residual surface flagged PAR2 after incubation with protease or vehicle for 60 minutes.

### Quantitative real-time PCR.

Total RNA was extracted with TRIzol LS reagent (Invitrogen), and cDNA was synthesized from 100 ng of total RNA with a LunaScript cDNA synthesis kit (New England Biolabs). Relative expression levels were determined by real-time PCR on a Bio-Rad CFX Connect or Real-Time System (CFX96 Real-Time System) using SYBR Green or the PowerUp SYBR Green kit (Thermo Fisher Scientific), and the primer sequences are listed in Supplemental Methods. *Rpl32* was used for normalization, and standard curves for each target gene were generated by pooled cDNA. Data are presented at a log_2_ scale as normalized expression to mean WT expression analyzed in parallel with mutant samples.

### Gut microbiome quantification.

The fecal samples isolated at the end of the antibiotic treatment experiment were used to quantify the microbiome content using PCR with site-specific primer of bacterial 16S gene as described previously (70). Bacterial DNA was isolated from the feces using a QIAamp PowerFecal DNA Kit (12830-50, QIAGEN), and PCR was carried out with 50 μg DNA template to quantify 16S DNA gene expression using bacterial primers (8F and 338R) and V6. Samples were run in duplicates, and 16S DNA gene expression was calculated as a relative ratio of Ct value of target normalized with Ct value of genomic DNA.

### GIP secretion assay in GLUTag cells.

GLUTag cells, originally derived by single-cell cloning of a proglucagon-SV40 large T antigen–induced tumor, were routinely maintained in high-glucose (25 mmol/L) DMEM (Gibco) supplemented with 10% FBS (44). For GIP secretion assay, cells were split into 24-well plates at a density of 200,000 cells per well in medium containing normal glucose (11 mmol/L). Forty-eight hours after plating, cells were rinsed with HBSS (Sigma-Aldrich), and cells were further incubated in medium containing 0.5% FBS and 5 mM glucose. GIP secretion was stimulated by addition of 25 mmol/L glucose for 2 hours. GIP concentrations in the culture supernatants were measured using GIP ELISA.

### Single-cell RNA sequencing of adult intestinal crypts and data analysis.

The mouse small intestines from 3 adult mice were dissected and cut into pieces of 4 mm^2^, placed into 50 mL conical tubes, and washed 3 times for 20 minutes with 25 mL of PBS/5 mM EDTA on a rocking plate at room temperature. Villi were mechanically dissociated by gentle shaking 3 times and trituration using a 25 mL pipette. Crypts were separated from the intestine by vigorous shaking 2 times and collected by centrifugation at 200*g* for 5 minutes, and crypts from 3 mice were mixed at equal ratios. Single-cell suspensions were stained with APC-conjugated anti–mouse CD326 (EpCAM) antibody (1:1,000; 17-5791-82, eBioscience) for 30 minutes at room temperature for fluorescence-activated cell sorting on a BD FACSAria III SORP cell sorter (100 μm nozzle). Living cells were gated by DAPI dye (Roche) exclusion. Fifty thousand single EpCAM^+^ cells were sorted in PBS supplemented with 0.4% BSA. Single-cell capturing, isolation of mRNA, and synthesis of cDNA used the BD Rhapsody Single-Cell Analysis System. Upon cDNA synthesis and exonuclease treatment, whole-transcriptome libraries were generated using a BD WTA Amplification Kit. The quantity of final libraries was assessed by Qubit Flex (Invitrogen) and the average size with a Bioanalyzer 2100 (Agilent). Sequencing was performed on a NovaSeq 6000 Plus (Illumina) at Novogene (Cambridge, United Kingdom).

The raw data were preprocessed according to the Illumina standard protocol, and reads were mapped to GENCODE M31. Counting and demultiplexing were performed using the BD Rhapsody Sequence Analysis Pipeline (v2.0), yielding around 10,000 called putative cells for each sample with a mean of around 45,000 aligned reads per cell. Pipeline output files were imported into Partek Flow software (Illumina). High-quality single cells were defined based on the following criteria: 2,000 ≤ genes per cell ≤ 10,000, mitochondrial UMI <25%. Single-cell counts were normalized by Partek Flow recommended normalization order (counts per million, Add:1 and log_2_). Two thousand genes with highest variance were used for linear dimensionality reduction (principal component analysis). The first 20 principal components were used for uniform manifold approximation and projection (UMAP) with the following parameters: local neighborhood size: 15; minimum distance: 0.3; distance metric: Euclidean; number of iterations: 100; initialize output values: spectral. Graph-based clustering was performed using the smart local moving (SLM) algorithm with the following parameters: resolution: 0.5; number of nearest neighbors: 30; number of random starts: 100; number of iterations per random start: 25; minimum cluster size: 50; sequential random starts: true; distance metric: Euclidean; nearest neighbor type: NN-descent. Marker genes for the clusters were identified using the ANOVA tool (Partek Flow). For enteroendocrine cell population, single-cell RNA sequencing data were analyzed in R using the Seurat package (v5). Cluster-specific marker genes were identified using FindAllMarkers, and results were exported as CSV files. To visualize expression patterns of selected genes across clusters, we generated dot plots using Seurat’s DotPlot function.

### Statistics.

Statistical analyses used GraphPad Prism 9 to 10. Statistical tests used included 1-way and 2-way ANOVA, 2-tailed *t* test, and Šidák’s multiple-comparison test. *P* values of less than 0.05 were considered significant.

### Study approval.

All animal experiments followed protocols approved by The Scripps Research Institute IACUC (protocol 09-0111), Johannes Gutenberg University Medical Centre Mainz (Landesuntersuchungsamt Koblenz, AZ 23-177-07/G14-1-55 and G 19-1-063), or the Institutional Animal Research Committee of Tongji Medical College (IACUC no. TJH-20230522).

### Data availability.

The RNA sequencing data presented in this article were deposited in the Gene Expression Omnibus (GEO) database under accessions GSE283949 and GSE277651.

All materials and reagents will be made available upon installment of a material transfer agreement.

## Author contributions

DK and SC performed the experiments and analyzed the data. CW, HW, MW, RW, PW, VVZ, and RG performed experiments. S Rachakonda, FM, and JJNP analyzed the bulk and single-cell RNA sequencing data. CR, S Reyda, CG, FS, and KS interpreted the data and supported experimental design. NS provided intestinal single-cell data and gut histology studies and supported experimental design. WR conceived and designed the study, interpreted the data, and wrote the manuscript. TM conceived and designed the study, conducted experiments, interpreted the data, and wrote the manuscript.

## Conflict of interest

The authors have declared that no conflict of interest exists.

## Funding support

This work is the result of NIH funding, in part, and is subject to the NIH Public Access Policy. Through acceptance of this federal funding, the NIH has been given a right to make the work publicly available in PubMed Central.

- Alexander Humboldt Foundation (to WR).
- National Heart, Lung, and Blood Institute HL60742 (to WR).
- Vascular biology research grants from the Boehringer Ingelheim Foundation for the collaborative research group “Novel and neglected cardiovascular risk factors: molecular mechanisms and therapeutics” (to TM and WR).
- Federal Ministry of Education and Research (Bundesministerium für Bildung und Forschung) grant 01EO1503 (to TM and WR).
- German Research Foundation (Project-ID 318346496; DFG-SFB1292 TP02 to WR; TP10 to CG and WR; TP19 to FM; and SO 1738/3-1 to NS).
- HW is supported by the Hubei Provincial Natural Science Foundation of China (Project-ID 2024EHA064).
- CR was awarded a Fellowship from the Gutenberg Research College at the Johannes Gutenberg University Mainz.
- DK is the recipient of a PhD stipend from the Institute of Molecular Biology Mainz.

## Supplementary Material

Supplemental data

Unedited blot and gel images

Supporting data values

## Version 1 03/17/2026

In-Press Preview

## Version 2 05/08/2026

Electronic publication

## Version 3 05/12/2026

Graphical abstract included
