# Supplementary material for: Epithelial TMPRSS2 impairs glucose homeostasis in obese mice by regulating ghrelin–GLP-1 receptor signaling pathway
Source: JCI Insight. 2026 Mar 17;11(9):e203211. doi: 10.1172/jci.insight.203211 (PMC13167073; doi:10.1172/jci.insight.203211)
Supplement: Supplemental data [file jciinsight-11-203211-s281.pdf]

Supplemental Data

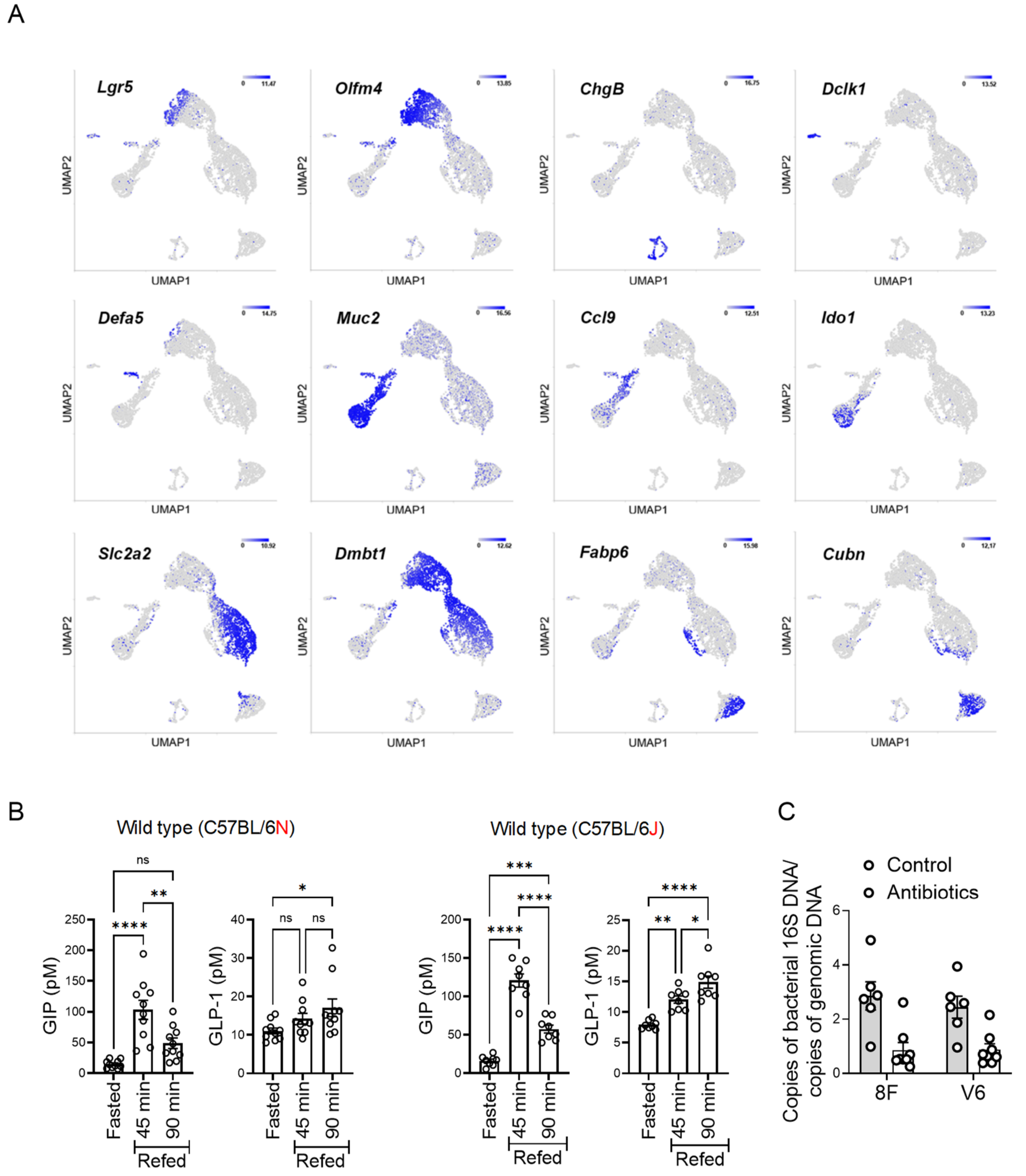

Supplemental Figure 1. Expression of characteristic cell type-specific genes in the UMAP of single cell RNA sequencing of intestinal epithelial cells. (A) *Lgr5*, *Olfm4:* intestinal stem cells (ISCs); *ChgB:* enteroendocrine cells (EECs); *Dclk1:* tuft cells; *Defa5:* Paneth cells; *Muc2*, *Ccl9* (early), *Ido1:* (mature) goblet cell populations; *Dmbt1* transient amplifying cells (TACs), *Slc2a2* (anterior), *Fabp6* and *Cubn* (posterior) enterocyte populations. (B) Plasma concentrations of postprandial GIP and GLP-1 in lean C57BL/6N and C57BL/6J mice measured at the indicated times after refeeding. Data represents mean ± SEM. (B) One-way ANOVA. *$P < 0.05$, **$P < 0.01$, ***$P < 0.001$. (C) Bacterial 16S DNA load in fecal pellets normalized to total mouse genomic DNA in the mice treated with or without antibiotics (n = 6/6).

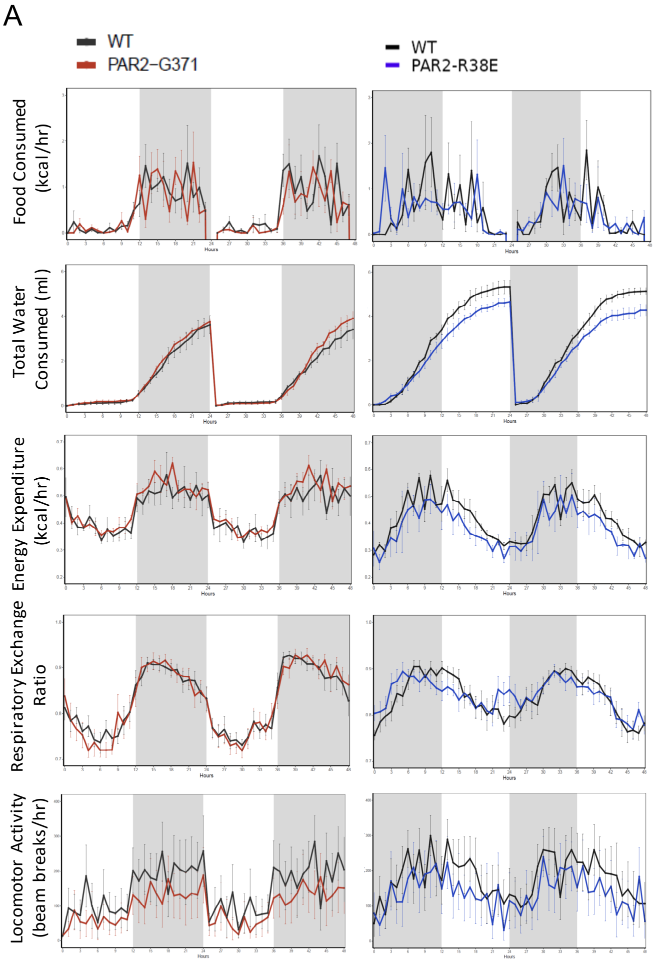

B

WT vs PAR2-G37I

Generalized linear model (GLM)

	Full day		Light		Dark	
**Effect**	**Mass**	**Group**	**Mass**	**Group**	**Mass**	**Group**
Food consumed (kcal/period)	0.5183	0.3944	0.6606	0.7301	0.0342*	0.2384
Water consumed (ml/period)	0.7961	0.0780	0.7461	0.4720	0.4406	0.2722
Energy expenditure (kcal/period)	0.5937	0.6826	0.5867	0.4040	0.6078	0.8780
Oxygen consumption (ml/hr)	0.5915	0.6892	0.5803	0.4252	0.6061	0.8728
Carbon dioxide production (ml/hr)	0.6015	0.6804	0.6397	0.3696	0.5997	0.9089

ANOVA

	Full day	Light	Dark
**Effect**	**Group**	**Group**	**Group**
Pedestrian locomotion(m)	0.4665	0.4485	0.5481
Total distance in cage (m)	0.3536	0.3221	0.4765
Respiratory exchange ratio	0.7842	0.8593	0.3660
Locomotor activity (beam breaks)	0.1028	0.0386*	0.2721

WT vs PAR2-R38E

Generalized linear model (GLM)

	Full day		Light		Dark	
**Effect**	**Mass**	**Group**	**Mass**	**Group**	**Mass**	**Group**
Food consumed (kcal/period)	0.8902	0.5705	0.1066	0.2767	0.4865	0.3962
Water consumed (ml/period)	0.5542	0.6066	0.2876	0.6704	0.3927	0.8774
Energy expenditure (kcal/period)	0.4473	0.9986	0.4239	0.9006	0.4689	0.9332
Oxygen consumption (ml/hr)	0.4604	0.9960	0.4291	0.9010	0.4885	0.9258
Carbon dioxide production (ml/hr)	0.3993	0.9478	0.3740	0.8087	0.4215	0.9755

ANOVA

	Full day	Light	Dark
**Effect**	**Group**	**Group**	**Group**
Pedestrian locomotion(m)	0.1058	0.0189*	0.2075
Total distance in cage (m)	0.0873	0.0178*	0.1680
Respiratory exchange ratio	0.8446	0.4498	0.6573
Locomotor activity (beam breaks)	0.1303	0.1146	0.1791

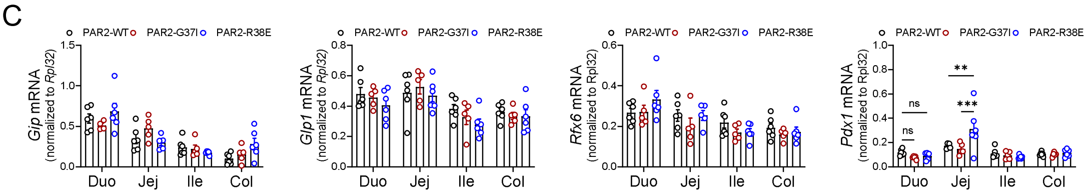

Supplemental Figure 2. Metabolic phenotyping of PAR2 mutant mice by indirect calorimetry. (A-B) Quantification of food intake (kcal/hr), water consumption, energy expenditure, respiratory exchange ratio and locomotor activity. (A) PAR2-G37I (n = 4/4) and PAR2-R38E (n = 3/3) were compared with WT mice. (B) Data analysis of individual metabolic parameters was performed using the online tool CalR; food intake and water consumption, energy expenditure was normalized to body weight (GLM: generalized linear model); respiratory exchange ratio and locomotor activity were determined independent of body weight (ANOVA). (C) Gene expression analysis of intestinal epithelial cells (IEC) isolated from fasted PAR2 mutant and strain-matched WT mice. Data represents mean ± SEM. (C) two-way ANOVA. *P < 0.05, **P < 0.01, ***P < 0.001.

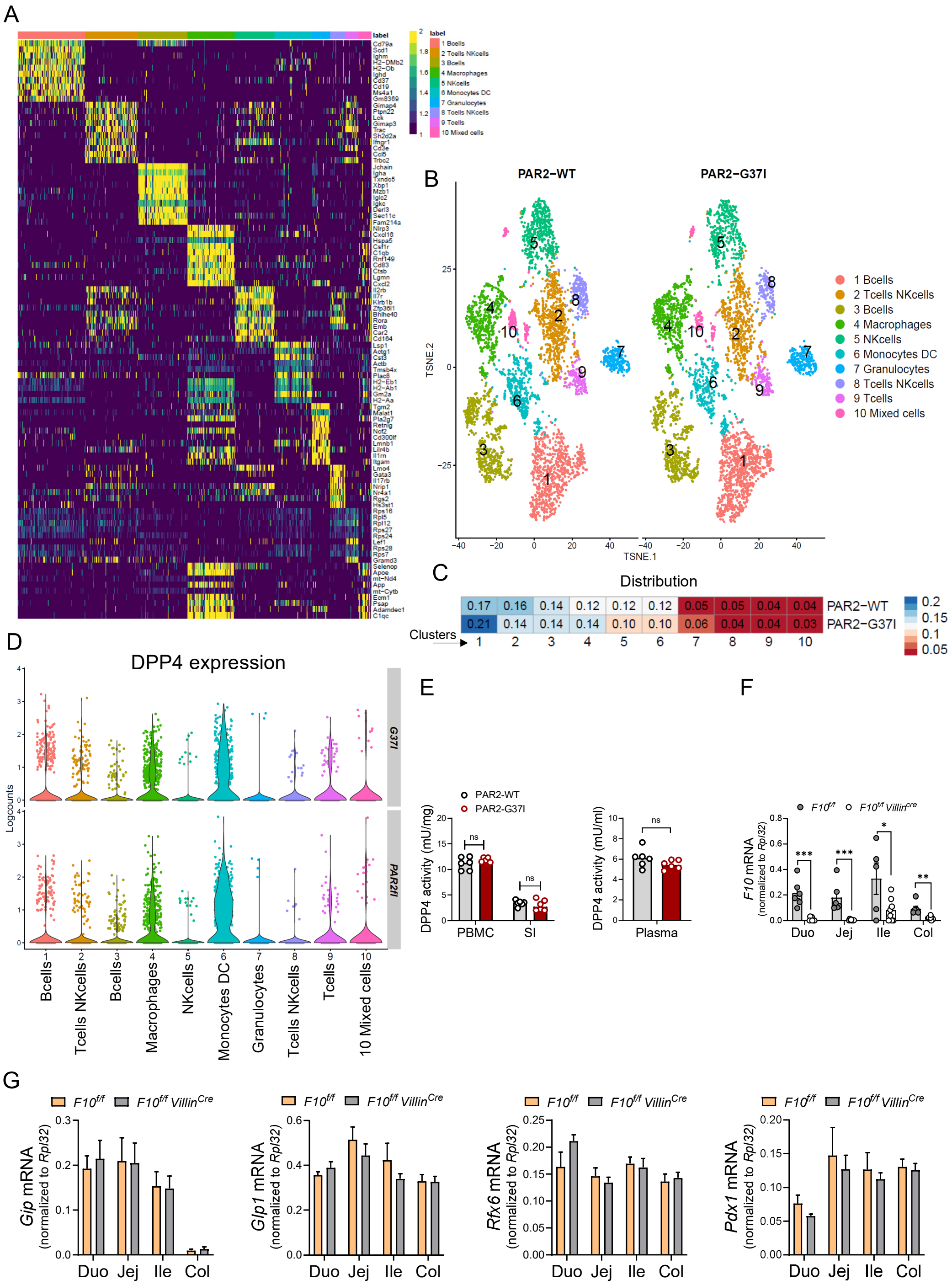

Clusters	1	2	3	4	5	6	7	8	9	10
PAR2-WT	0.17	0.16	0.14	0.12	0.12	0.12	0.05	0.05	0.04	0.04
PAR2-G37I	0.21	0.14	0.14	0.14	0.10	0.10	0.06	0.04	0.04	0.03

Supplemental Figure 3. Characterization of lean PAR2-G37I and *F10^f/f^ Villin^Cre^* mice (A) Single cell RNA sequencing of CD45$^+$ lamina propria immune cells isolated from the small intestine. Heat map showing top 10 cluster defining genes for cells isolated from lean, aged-matched PAR2-G37I mutant and WT mice; n = 4/4 (B) t-SNE (t-distributed Stochastic Neighbor Embedded) plot showing immune cell clusters. (C) Heat map showing relative abundance of immune cells clusters in PAR2-G37I versus WT mice. (D) DPP4 gene expression across immune cell clusters analyzed by the single cell RNA sequencing of CD45-selected cells isolated from the intestinal lamina propria. (E) DPP4 activity in peripheral blood monocytes (PBMCs), small intestine and plasma from lean mice fasted overnight and refed for 1 hour. Mean ± SEM; n $\geq$ 5 mice/group. (F-G) Characterization of *F10$^{f/f}$ Villin^{Cre}* mice. Gene expression analysis of IECs isolated from lean *F10$^{f/f}$ Villin^{Cre}* mice and littermate control *F10$^{f/f}$* mice; n = 6/6. Data represents mean ± SEM. (E-F) two-tailed t test. *P < 0.05, **P < 0.01, ***P < 0.001.

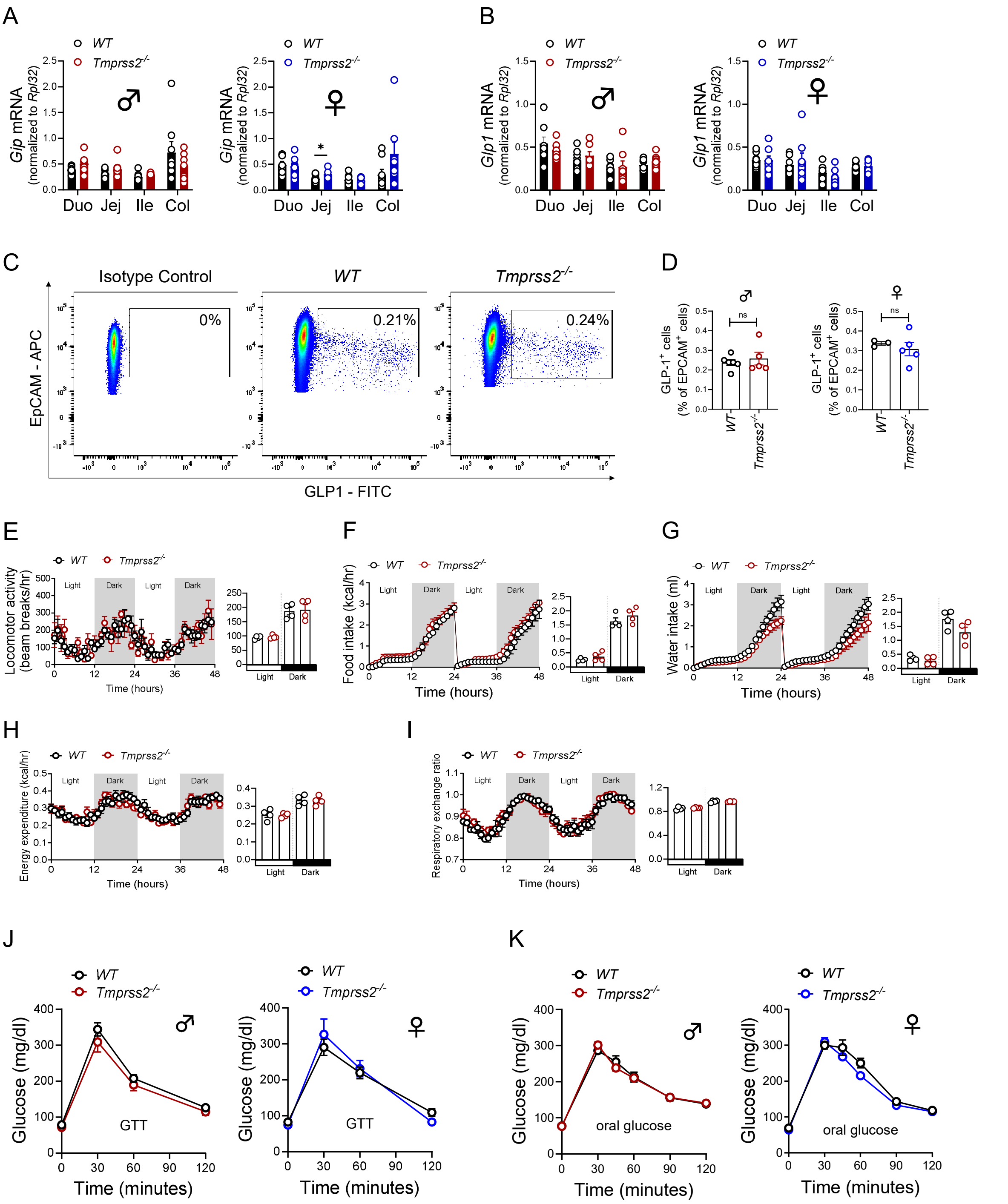

Supplemental Figure 4. Characterization of lean *Tmprss2$^{-/-}$* mice. (A) GIP and (B) GLP-1 mRNA expression in IECs isolated from *Tmprss2$^{-/-}$* and age and strain matched WT mice. (C) Flow cytometry gating strategy for the detection of GLP-1^{+} cells within EPCAM^{+} IECs. (D) Quantification EPCAM^{+}/GLP-1^{+} IEC. (E) Locomotor activity, (F) food intake in (kcal/hour), (G) cumulative water intake, (H) energy expenditure and (I) respiratory exchange ratio determined for lean *Tmprss2$^{-/-}$* and WT mice (n = 4/4). Metabolic data was analyzed with CalR and energy expenditure was normalized to body weight. (J) GTT following intraperitoneal glucose administration and (K) oral glucose challenge in lean male and female *Tmprss2$^{-/-}$* and WT mice. Data represents mean ± SEM. (A, B, D, E-I) two-tailed t test. (J, K) two-way ANOVA. *P < 0.05, **P < 0.01, ***P < 0.001.

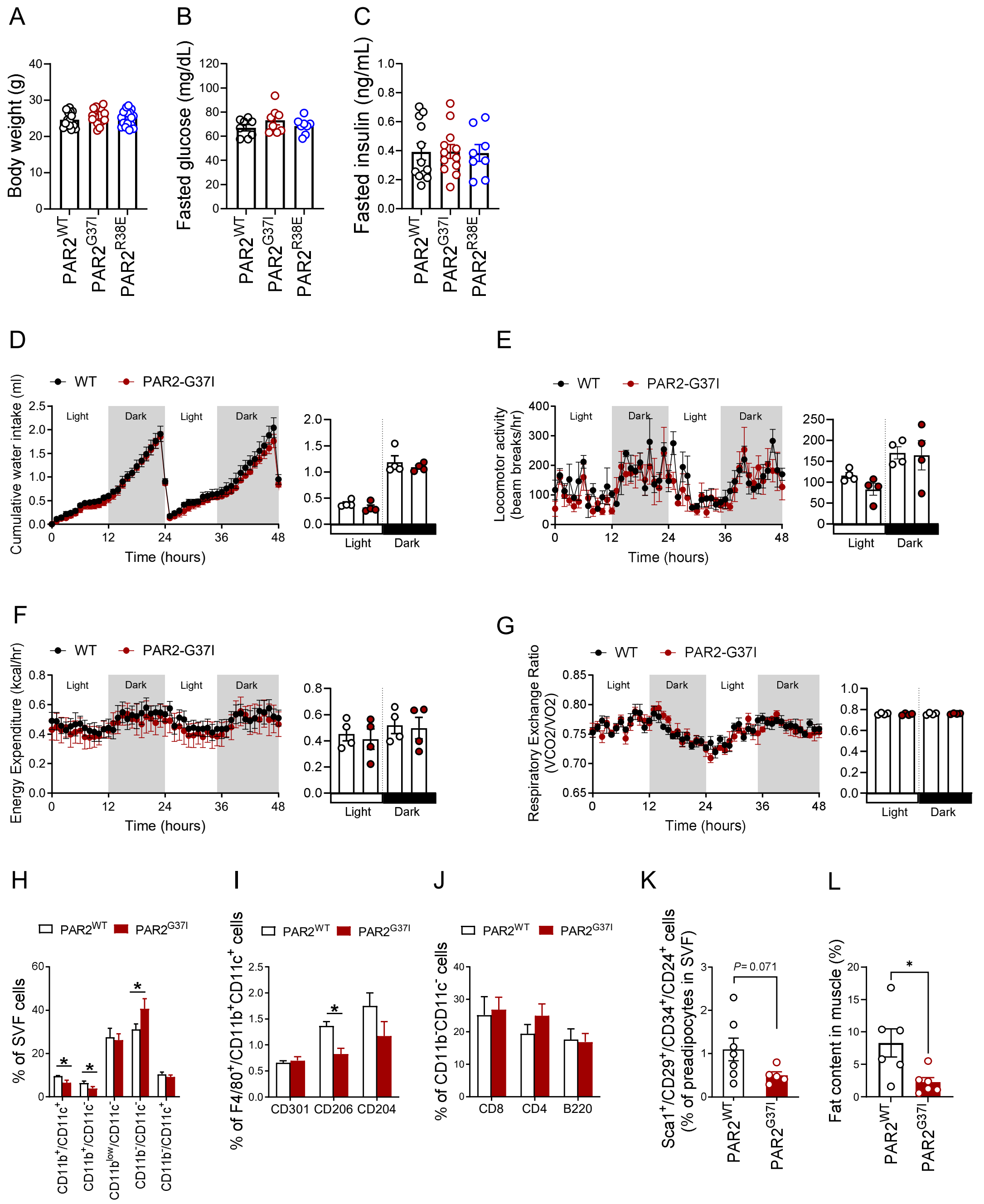

Supplemental Figure 5. Metabolic and immune phenotyping of obese PAR2 mutant and WT mice. (A) Body weights, (B) fasted plasma glucose levels, and (C) insulin concentrations in male PAR2 mutant and WT mice aged 10-12 weeks on NCD. (D-G) Metabolic phenotyping of male PAR2-G37I and WT mice on HFD for 14 weeks (n = 4/4). Phenotyping of individual mice in metabolic cages measured cumulative water intake (D), locomotor activity (E), energy expenditure (F), and the respiratory exchange ratio (G). Metabolic data was analyzed with CalR and food intake, water intake and energy expenditure were normalized to the body weight. (H-L) Immunophenotyping of adipose tissue stromal vascular fractions (SVF) isolated from mice on HFD for 14 weeks. (H) Total myeloid cells ($CD11b^+$) and non-myeloid stromal cells ($CD11b^-$) were further analyzed to determine the percentage of pro-inflammatory cells ($CD11c^+$). (I) Expression of markers for alternative macrophage activation CD301, CD206 and CD204 on $F4/80^+/CD11b^+/CD11c^+$ cells. (J) $CD8^+$ and $CD4^+$ T and $B220^+$ B cells within the $CD11b^-/CD11c^-$ cells. (K) Quantification of $Sca1^+/CD29^+/CD34^+/CD24^+$ preadipocytes in the SVF (L) Fat content in muscle analyzed by Echo-MRI. Data represents mean ± SEM. (A-C) one-way ANOVA, (H-L) two-tailed t test. *P < 0.05, **P < 0.01, ***P < 0.001.

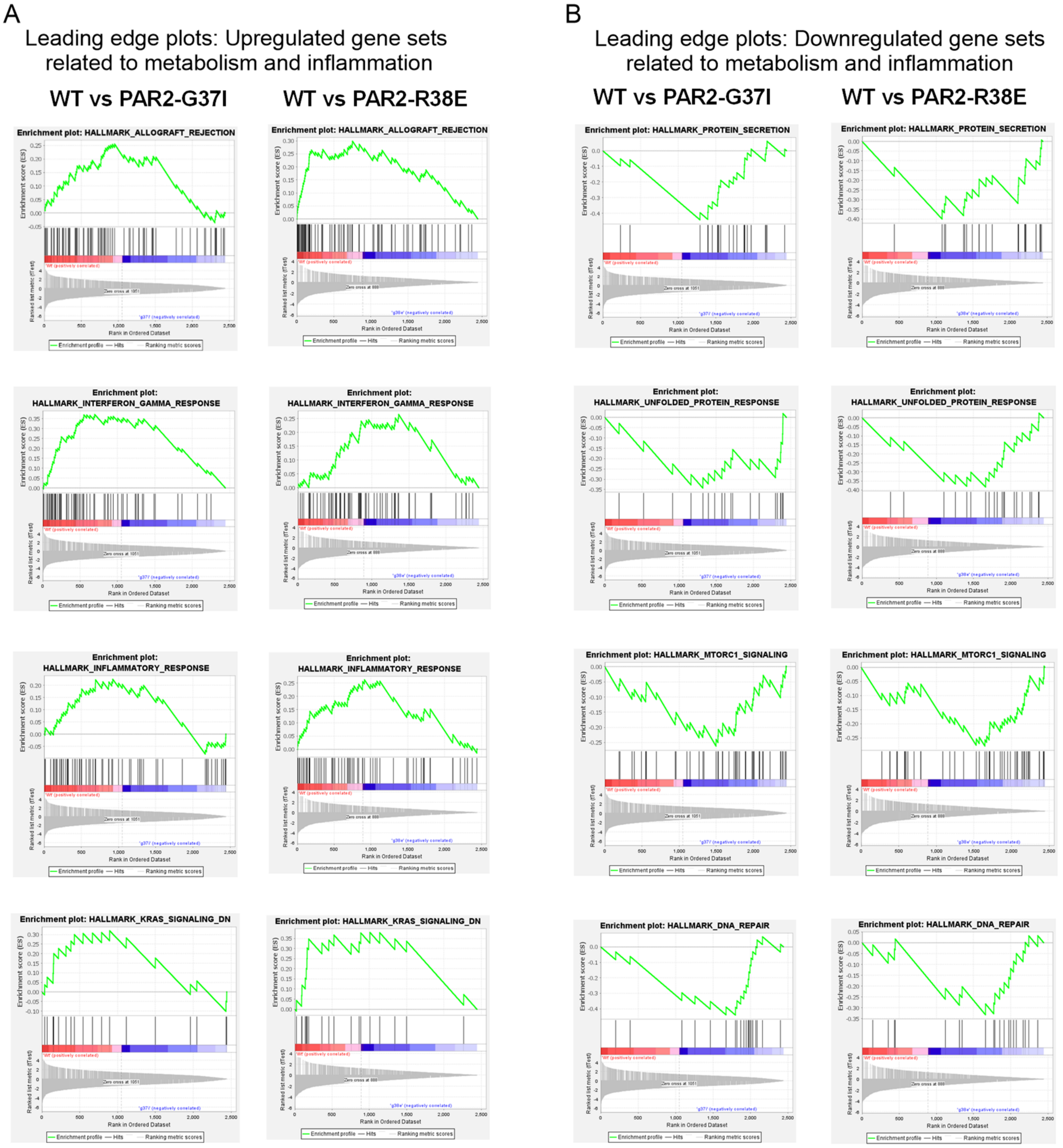

Supplemental Figure 6. Pathway analysis of CD11b+ CD11c+ ATM from PAR2-G37I, PAR2-R38E, and WT mice on HFD. (A-B) Leading edge plots showing differentially regulated gene sets related to upregulated (A) and downregulated (B) hallmark pathways in CD11b+CD11c+ ATM from PAR2 mutant and WT mice on a HFD for 16 weeks. Differentially regulated gene expression patterns and associated hallmark pathways were largely overlapping in ATM derived from HFD-fed PAR2-mutants G37I and R38E mice.

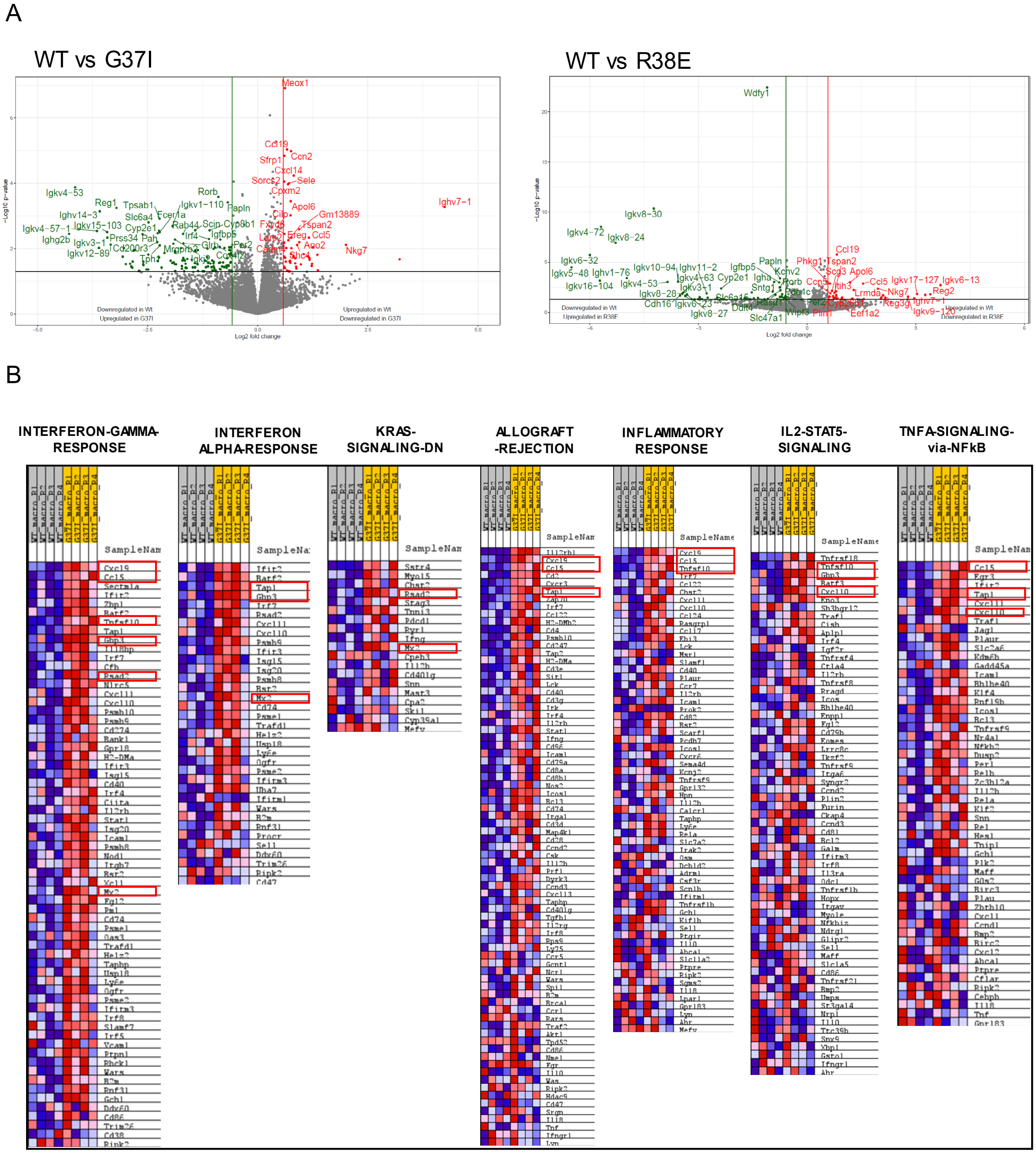

Supplemental Figure 7. Transcriptional profiling of the SVF in obese PAR2-G37I, PAR2-R38E and WT mice. (A) Volcano plots of differentially regulated genes in endothelial cells of PAR2-G37I and PAR2-R38E mice. (B) Heat maps showing pathway-specific differentially regulated gene sets in $CD11b^+CD11c^+$ ATM isolated from the SVF of HFD fed PAR2-G37I mutant and WT mice. Characteristic markers used for quantitative real-time polymerase chain reaction (qRT-PCR) analysis in Fig. 4F are highlighted in red.

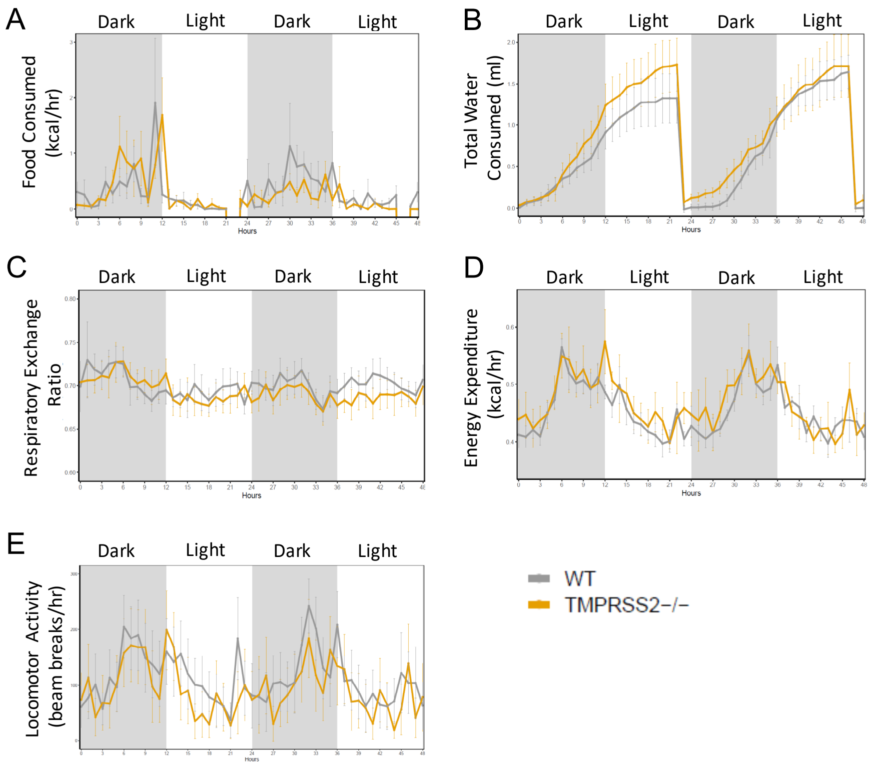

F

Generalized linear model (GLM)

	Full day		Light		Dark	
**Effect**	**Mass**	**Group**	**Mass**	**Group**	**Mass**	**Group**
Food consumed (kcal/period)	0.3307	0.2470	0.5471	0.5267	0.9896	0.9326
Water consumed (ml/period)	0.5675	0.5976	0.2626	0.6656	0.3533	0.9292
Energy expenditure (kcal/period)	0.7427	0.5903	0.7550	0.6151	0.7341	0.5728
Oxygen consumption (ml/hr)	0.7614	0.6030	0.7663	0.6214	0.7853	0.5897
Carbon dioxide production (ml/hr)	0.6532	0.6120	0.6392	0.6371	0.6659	0.5966

ANOVA

	Full day	Light	Dark
**Effect**	**Group**	**Group**	**Group**
Pedestrian locomotion(m)	0.2490	0.2847	0.1965
Total distance in cage (m)	0.3451	0.3831	0.3215
Respiratory exchange ratio	0.5320	0.5045	0.5866
Locomotor activity (beam breaks)	0.1490	0.1055	0.2378

Supplemental Figure 8. Metabolic phenotyping of obese TMPRSS2$^{-/-}$ mice. (A) Food intake (kcal/hr), (B) water intake, (C) respiratory exchange ratio, (D) energy expenditure, and (E) locomotor activity of *Tmprss2*$^{-/-}$ and WT mice on a HFD for 14 weeks. (F) Summarized data of three consecutive days was analyzed with CalR. The table listed under 'GLM' has p-values from a model including the selected mass as a covariate. The table listed under 'ANOVA' has p-values from a model including only group as predictor variable. The tables display p-values corresponding to group effect (ANOVA) along with the mass and interaction effects as necessary (GLM).

Supplementary Materials

REAGENT or RESOURCE	SOURCE	IDENTIFIER
**Chemicals, Peptides, and Recombinant Proteins**		
Ampicillin	Roth	K029.1
Bacitracin	Sigma	11702
Bovine serum albumin (BSA)	Sigma	A7030
Brij-35	Sigma	B4184
BSA-octanoate saturated fatty acid complex	Cayman	34933
Ceftazidime hydrate	Sigma	A6987
Ciprofloxacin	Sigma	PHR1167
Collagenase A	Roche	11088793001
DAPI	Roche	11088793001
Diprotin A	Enzo	ALX-260-036-M025
Dispase II	Sigma	D4693
Dithiothreitol (DTT)	PanReac	A1101
D-Lys^3-GHRP-6	Sigma	G4535
Ethylenediaminetetraacetic acid (EDTA) 0.5 M Solution	Invitrogen™	15575-038
Fetal calf serum (FCS) Sera Plus	Pan-Biotech	P30-3702
Ficoll®-PaqueTM Premium 1.084	GE healthcare	17-5446-52
Glucose	Sigma	G8270
HEPES solution	Sigma	H0887
Lipofectamine^{TM} 2000	Invitrogen	11668-027
Luna Script RT Super Mix	New England Biolabs	E3010L
Luna Universal qPCR Master Mix	New England Biolabs	M3003E
MEM Non-essential amino acid solution (100X)	Sigma	M7145
Metronidazole	Sigma	M3761
Neomycin trisulfate salt hydrate	Sigma	N6386
NP-40 Detergent Solution	Thermo Fischer	85124
Penicillin-Streptomycin	Sigma	P0781
Phosphatase inhibitor tablets	Roche	04906837001
Protease inhibitor cocktail tablets	Roche	04693159001

RNA later® solution	Invitrogen	AM7020
Trypsin/ EDTA	Gibco	25200-056
Vancomycin	Vanco-saar	27776
Vectashield	Vector Labs	H-1000-10
β-mercaptoethanol	Sigma	M3148
**Antibodies and probes**		
Anti-mouse CD11b-efluor450 (M1/70)	eBioscience	Cat #48-0112-82
Anti-mouse CD11b-PerCP-Cy5.5(M1/70)	eBioscience	Cat #45-0112-82
Anti-mouse CD11c-APC (N418)	eBioscience	Cat #17-0114-82
Anti-mouse CD11c-PE-Cy7 (N418)	eBioscience	Cat #25-0114-82
Anti-mouse F4/80-PE (BM8)	eBioscience	Cat #12-4801-82
Anti-mouse F4/80-FITC (BM8)	eBioscience	Cat #11-4801-82
Anti-mouse F4/80-efluor450 (BM8)	eBioscience	Cat #48-4801-82
Anti-mouse CD301-PE-Cy7 (LOM-8.7)	Biolegend	Cat #145609
Anti-mouse CD206-PercP-Cy5.5 (CO68C2)	Biolegend	Cat #141716
Anti-mouse CD204-PE (REA 148)	Miltenyi Biotec	130-102-285
Anti-mouse CD8-APC (GK1.5)	eBioscience	Cat #17-0081-82
Anti-mouse CD4-PE (RM4-5)	eBioscience	Cat #12-0041-82
Anti-mouse B220-PE-Cy7 (RA3-6B2)	eBioscience	Cat #25-0452-82
Anti-mouse Sca1-PE (D7)	eBioscience	Cat #12-5981-82
Anti-mouse CD29-PercP-eFluor710 (eBioHmb1-1)	eBioscience	Cat #46-0291-82
Anti-mouse CD34-efluor450 (RAM34)	eBioscience	Cat #48-0341-82
Anti-mouse CD24-PE-Cy7 (M1/69)	eBioscience	Cat #25-0242-82
Anti-mouse EPCR-PE (eBio1560)	eBioscience	Cat #12-2012-82
Anti-mouse CD105-PE (MJ7/18)	eBioscience	Cat #12-1051-82
Anti-mouse CD45-FITC (30-F11)	eBioscience	Cat #11-0451-82
Anti-mouse CD326 (EpCAM) APC Clone G8.8	eBioscience	17-5791-82
Anti-mouse TMPRSS2 (H-4) monoclonal Ab.	Santa Cruz Biotechnology	Sc-515727
Anti-mouse PAR2 rabbit polyclonal	Custom	Custom
Anti-GLP-1 Recombinant Rabbit monoclonal Ab	Thermo Fischer	MA5-42868

Phospho-IGF-I Receptor beta (Tyr1135/1136)/Insulin Receptor beta (Tyr1150/1151) (19H7)-Rabbit mAb	Cell signaling Technology	3024
Insulin Receptor beta (4B8) Rabbit-mAb	Cell signaling Technology	3025
Phospho-p44/42 MAPK (Erk1/2) (Thr202/Tyr204) (D13.14.4E) Rabbit-mAb	Cell signaling Technology	4370
p44/42 MAPK (Erk1/2) (137F5) Rabbit Monoclonal Antibody	Cell signaling Technology	4695
beta-Actin (8H10D10) Mouse-mAb	Cell signaling Technology	3700
Anti-rabbit IgG, HRP-linked Antibody	Cell signaling Technology	7074
Anti-mouse IgG, HRP-linked Antibody	Cell signaling Technology	7076
Donkey anti-Rabbit IgG (H+L) Alexa Fluor Plus 488	Invitrogen	A32790
Goat anti-mouse IgG (H+L) Alexa594	Abcam	Ab150116
**Commercial Assays**		
BCA Protein Assay Kit	Pierce™	23225
BD Mouse immune Single-Cell Multiplexing Kit	Becton Dickinson	633793
BD Rhapsody™ Cartridge Kit	Becton Dickinson	633733
BD Rhapsody™ Cartridge Reagent Kit	Becton Dickinson	633731
BD Rhapsody™ cDNA Kit	Becton Dickinson	633773
DPP4 Activity Assay Kit	Sigma	MAK088-1KT
Mouse CD45 Microbeads	Miltenyi Biotec	130-052-301
Mouse CD11c Microbeads	Miltenyi Biotec	130-125-835
Mouse Insulin ELISA Kit	Alpco	80-INSMS-E01
Mouse Lamina Propria Dissociation Kit	Miltenyi Biotec	130-097-410
Mouse total GIP Elisa Kit	Crystal Chem	81527
Mouse total GLP-1 Elisa Kit	Crystal Chem	81508
QIAamp Power Fecal DNA Kit	Qiagen	12830-50
Rat/Mouse total Ghrelin Elisa Kit	Merck	EZRGRT-91K
Tissue lyser II	Qiagen	

**Media/Solutions**		
Dulbecco's Modified Eagles Medium (1X DMEM)	Gibco	21063
Dulbecco's Phosphate Buffered Saline (1X DPBS), with Ca^{2+} and Mg^{2+}	Gibco	14040
DMEM/F-12 (1:1) (1X) + GlutaMax™	Gibco	31331-028
Dulbecco's Phosphate Buffered Saline (1X PBS)	Sigma-Aldrich	D8537
Fetal calf serum (FCS) Sera Plus	Pan-Biotech	P30-3702
ACK Lysing Buffer Lonza™	Lonza	10-548E
Hank's buffered salt solution (1X HBSS)<br>With Ca^{2+} and Mg^{2+}, without phenol red;<br>Without Ca^{2+} and Mg^{2+}, with phenol red	Sigma<br>Gibco	55037C<br>14170
Opti-MEM® I (1X)	Gibco	31985-062
RIPA Buffer (10X)	Cell Signaling	9806S
**Cell Lines**		
CHO-K1 cells	ATCC	
GLUTag cell line	Obtained from Daniel J Druker	Lunenfeld-Tanenbaum Research Institute, Toronto, Ontario, Canada
**Software and Algorithms**		
CFX Maestro™	Bio-Rad	
FlowJo software v10.10.0	BD Biosciences	
CalR (Web application for indirect calorimetry analysis)	Free-Online access	https://calrapp.org/ (ref.1)
Microsoft Office	Microsoft Corporation	
Scientific Image and Illustration Software	BioRender	www.biorender.com
Statistical Analysis	GraphPad Prism	
EndNote X9	Clarivate™ Analytics	
**Rodent diet**		
High fat diet (HFD)	Research Diets	D12492

Normal chow diet (NCD)	Ssniff Diets	

**Mouse Primers**	**Sequences**
m CCL5 fw	5'-GAGTGACAAACACGACTGCAAGAT-3'
m CCL5 rev	5'-CTGCTTTGCCTACCTCTCCCT-3'
m CXCL10 fw	5'-GCTCAGGCTCGTCAGTTCTA-3'
m CXCL10 rev	5'-AGATGGTGGTTAAGTTCGTGCT-3'
m CXCL9 fw	5'-TTTTCCTTTTGGGCATCATCTT-3'
m CXCL9 rev	5'-AGCATCGTGCATTCCTTATCACT-3'
m FX fw	5'-TTCCGGATGAACGTGGCCCCT-3'
m FX rev	5'-ATGCGTGCGTCCAAAACCGCT-3'
m Gbp3 fw	5'-CTGACAGTAAATCTGGAAGCCAT-3'
m Gbp3 rev	5'-CCGTCCTGCAAGACGATTCA-3'
m GHRL fw	5'-GAAGCCACCAGCTAAACTGCAG-3'
m GHRL rev	5'-CTGACAGCTTGATGCCAACATCG-3'
m GIP fw	5'-GTGGCTTTGAAGACCTGCTC-3'
m GIP rev	5'-TTGTTGTCGGATCTTGTCCA-3'
m GLP-1 fw	5'-TGAAGACAAACGCCACTCAC-3'
m GLP-1 rev	5'-TGACGTTTGGCAATGTTGTT-3'
m PDX-1 fw	5'-GAAATCCACCAAAGCTCACG-3'
m PDX-1 rev	5'-CGGGTTCCGCTGTGTAAG-3'
m pIgR fw	5'-ACCAGAGGGTCATCCCATGT-3'
m pIgR rev	5'-AAAGGCCCGGGATTTGGAAT-3'
m RFX6 fw	5'-TCAGCTCCAACACATTGCTC-3'
m RFX6 rev	5'-CTCTGCACGGTGAGCATAGA-3'
m RPL32 fw	5'-CCTCTGGTGAAGCCCAAGATC-3'
m RPL32 rev	5'-TCTGGGTTTCCGCCAGTTT-3'
m RSAD2 fw	5'-TGCTGGCTGAGAATAGCATTAGG-3'
m RSAD2 rev	5'-GCTGAGTGCTGTTCCCATCT-3'
m TAP1 fw	5'-GGACTTGCCTTGTTCCGAGAG-3'
m TAP1 rev	5'-GCTGCCACATAACTGATAGCGA-3'
m Tnfsf10 fw	5'-GAATGGAGAGCTGGTCATCGAG-3'
m Tnfsf10 rev	5'-GATCCGGATAGCTGGTGTACTTGTAG-3'
**Bacterial Primers**	**Sequences**
8F	5'-AGAGTTTGATCCTGGCTCAG-3'
338R	5'-TGCTGCCTCCCGTAGGAGT-3'

V6 F	5'-AGGATTAGATACCCTGGTA-3'
V6 R	5'-CRRCACGAGCTGACGAC-3'

Supplemental Methods

Analysis of metabolic data

Summarized data of consecutive days was analyzed with CalR (1). CalR implements generalized linear model (GLM) for the analysis of metabolic variables (food consumption, water consumption, energy expenditure, V_{O2} and V_{CO2} consumption). The analysis is conducted on subsets of the data categorized by time of day. Each row represents the results of statistical test for which the metabolic variable is being predicted by the group (and mass). The table listed under 'GLM' has p-values from a model including the selected mass as a covariate. The table listed under 'ANOVA' has p-values from a model including only group as predictor variable.

Isolation of immune cells from Lamina propria for single cell RNA-sequencing

The protocol for immune cells isolation from the laminar propria is derived from the IEC isolation protocol with some modifications. The Peyer's patches from the intestine were removed, prior to the longitudinal dissection of the intestinal segments. Tissue digestion was performed using Mouse Lamina Propria Dissociation Kit from Miltenyi Biotec (130-097-410).

DPP4 activity assay

DPP4 activity was measured in 5µl of plasma, PBMC cell lysate, and small intestine (diluted 1:8 with assay buffer) using fluorometric DPP4 activity assay kit (Sigma: MAK088). Briefly, tissue samples collected in 1 ml of lysis buffer containing (50mM Tris HCL, pH 8.0, 1mM EDTA, 10%glyerol, 0.02% Brij-35, protease inhibitor (Roche)) were homogenized using the TissueLyser II (Qiagen) system and kept on ice for 30 min followed by centrifugation at 8000g for 10 min. Next, total protein concentration in each sample was determined using BCA protein assay kit (Pierce: 23325). After protein estimation, the reaction was initiated using a non-fluorescent synthetic substrate (H-glycyl-prolyl-AMC) that cleaves by endogenous DPP4

present in the sample to form a fluorescent product 7-Amino-4-Methyl Coumarin (AMC). The fluorescent signal was then measured in the SpectraMAX i3 plate reader with 380/460 nm excitation/emission spectra. The DPP4 activity in the unknown sample was determined using AMC standard curve as the rate of production of AMC per min per ml from the substrate. The DPP4 activity is reported as microunits/ml and was normalized to total protein content in case of tissue/cell lysate.

ATM isolation and FACS analysis

For the isolation of adipose tissue macrophages (ATM), epididymal fat pads were isolated from the mice fed HFD for 14-16 weeks. The dissected epididymal fat pads were minced with scissors and placed in 50 ml conical tube containing 20 ml of collagenase A solution (1 mg/ml) (Roche: 11088793001). The samples were digested in an incubator shaker at 37°C for 25 minutes. The digested fractions were filtered through the 100 µm sterile filters (Falcon cell strainer) into a new 50 ml sterile tube. The filtered samples were further centrifuged at 500g for 5 minutes to separate floating adipocytes from the SVF pellet. The supernatant was discarded and the SVF pellet was resuspended in RBC lysis buffer (ACK Lysing Buffer, Lonza:10-548E) and incubated for 3 to 5 minutes in a new sterile tube. After the lysis of the RBCs, the cells were washed twice with PBS supplemented with 0.5% BSA and used further for the enrichment of ATM by immunomagnetic bead separation. Anti-CD11b and anti-CD11c beads were used for immunomagnetic separation of the proinflammatory ATM for quantitative polymerase chain reaction (qPCR) analysis. For gene expression analysis, the CD11b^{high}-Cd11C^{high} double positive ATM were further enriched by FACS sorting prior to next generation sequencing (NGS). Antibody conjugated immunomagnetic beads for CD45, CD11c, Cd11b were obtained from Miltenyi Biotec. The anti-mouse antibodies: CD11b-efluor450 (M1/70); CD11b-PerCP-Cy5.5(M1/70); CD11c-APC (N418); CD11c-PE-Cy7 (N418); F4/80-PE (BM8); F4/80-FITC (BM8); F4/80-efluor450 (BM8); CD8-APC (GK1.5); CD4-PE (RM4-5); B220-PE-Cy7 (RA3-6B2); Sca1-PE (D7); CD29-PercP-eFluor710 (eBioHmb1-1); CD34-efluor450 (RAM34); CD24-PE-Cy7 (M1/69); EPCR-PE (eBio1560); CD105-PE (MJ7/18); CD45-FITC

(30-F11) were obtained from eBioscience. Anti-mouse CD301-PE-Cy7 (LOM-8.7) and Anti-mouse CD206-PercP-Cy5.5 (CO68C2) were from Biolegend. Anti-mouse CD204-PE (REA 148; 130-102-285); Mouse Cd45 microbeads, Cd11b and CD11c microbeads from Miltenyi Biotec.

Bioinformatics analysis of bulk and single-cell RNA sequencing

Quality control on the sequencing data was performed with the FastQC tool (version 0.11.9), https://www.bioinformatics.babraham.ac.uk/projects/fastqc/). RNA sequencing reads were aligned to the ENSEMBL Mus_musculus.GRCm38 reference genome. The corresponding annotation (ENSEMBL v76) was also retrieved from ENSEMBL FTP website. The STAR aligner (version 2.6.1a) was used to perform mapping to the reference genome. Alignments were processed with the feature-Counts function of the R-subread package (version 1.32.4) using the annotation file also used for supporting the alignment. The exploration, modeling, and interpretation of the expression data follows the protocols defined by Ludt et al (2022) (2). Differential expression analysis was performed with DESeq2 package (version 1.22.2), setting the false discovery rate (FDR) cutoff to <0.05 and volcano plots were drawn for genes that were statistically significant genes. Accurate estimation of the effect sizes (described as log2 fold change) was performed using the apeglm shrinkage estimator (version 1.4.2). Unsupervised hierarchical clustering was performed on genes with a differential expression of ±0.58 log2FC (1.5-fold change) using Euclidean distance and the ward.D2 method via the Complex Heatmap package in R. Further analyses such as functional enrichment results were further processed with the Gene Tonic package for visualization and summarizing.

The genes in the RNA seq data were mapped to the fifty Hallmark gene sets (MSigDB 2023.2Mm) representing well-defined cellular signaling pathways in mouse models. The genes were sorted by rank-metric based on the respective p-values and the sign of the log fold-change (-log10(p-value) *sgn(logFC) obtained from differentially expressed genes (DEG) analysis. Accordingly, the genes with positive and negative values represent upregulated and downregulated genes respectively. The normalized enrichment score (NES) was obtained

using 1000 gene set permutations and the threshold for significant enrichment was defined as a p-value of <0.05 and an FDR of <0.25. The NES indicates the degree to which gene set is overrepresented at the top or bottom of the ranked list of genes in this gene set.

References

1. Mina AI, LeClair RA, LeClair KB, Cohen DE, Lantier L, and Banks AS. CalR: A Web-Based Analysis Tool for Indirect Calorimetry Experiments. *Cell Metab.* 2018;28(4):656-66 e1.
2. Ludt A, Ustjanzew A, Binder H, Strauch K, and Marini F. Interactive and Reproducible Workflows for Exploring and Modeling RNA-seq Data with pcaExplorer, Ideal, and GeneTonic. *Curr Protoc.* 2022;2(4):e411.
