# Supplementary figures and images for: Epithelial TMPRSS2 impairs glucose homeostasis in obese mice by regulating ghrelin–GLP-1 receptor signaling pathway
Source: JCI Insight. 2026 Mar 17;11(9):e203211. doi: 10.1172/jci.insight.203211 (PMC13167073; doi:10.1172/jci.insight.203211)

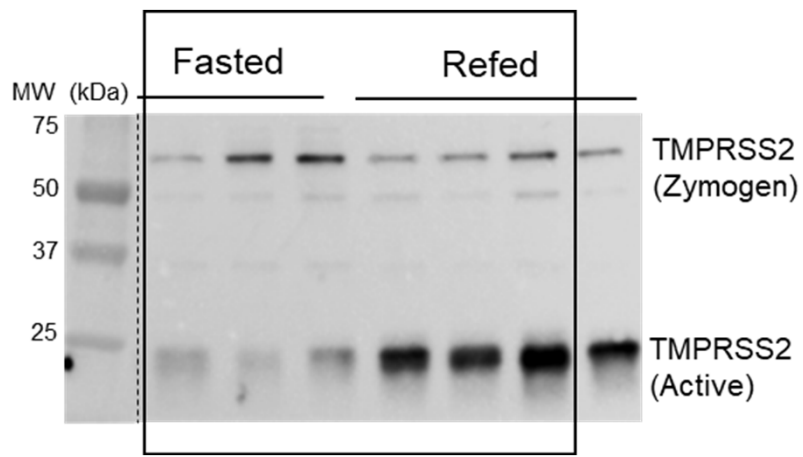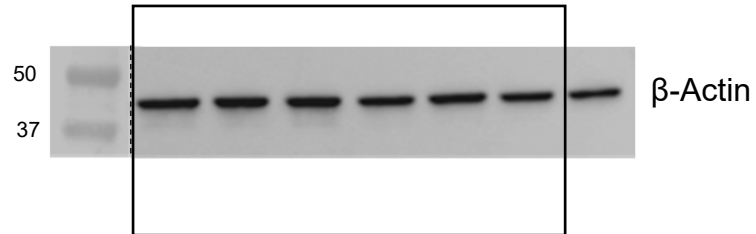

Figure 5H (left panel): Unedited western blot

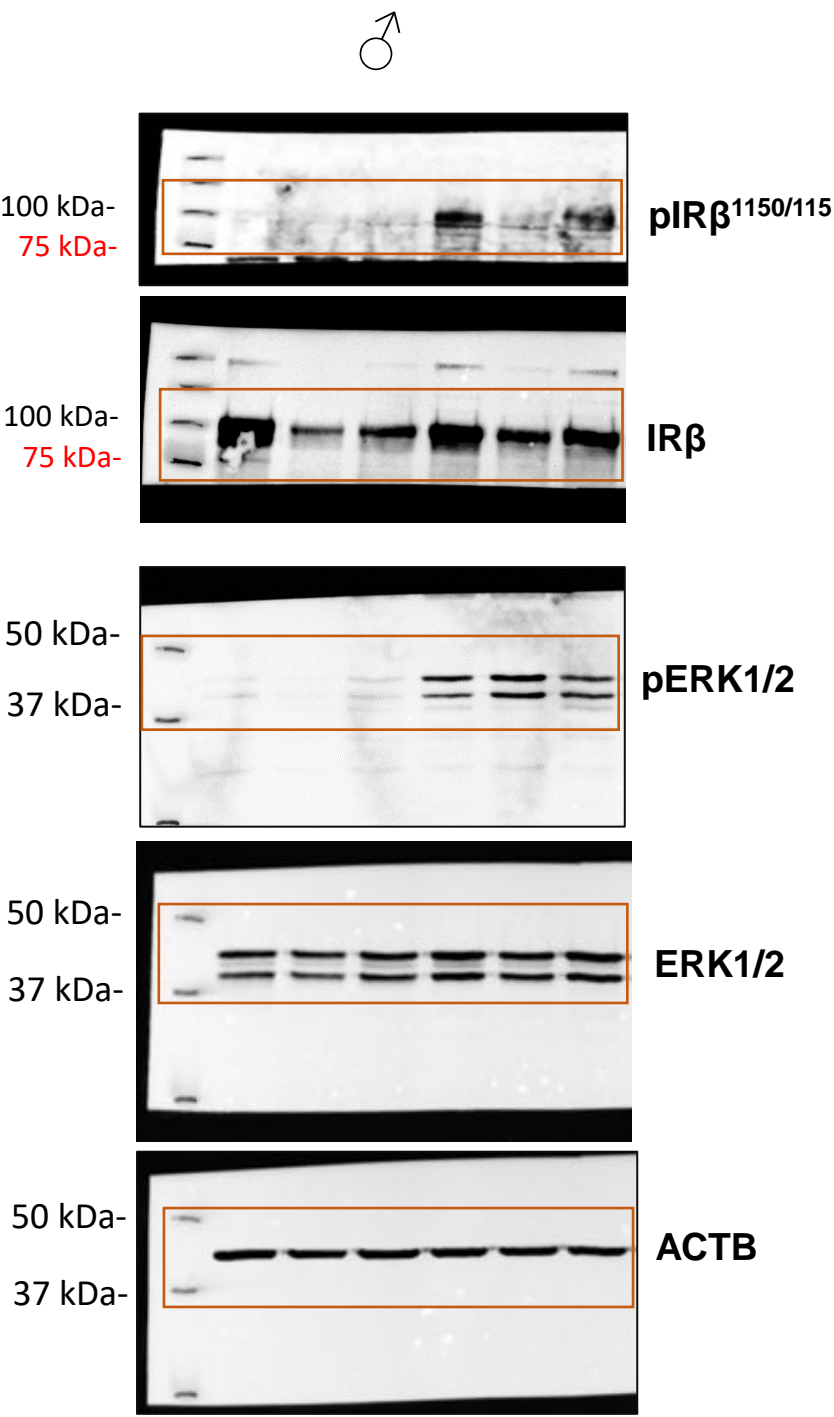

Figure 5H (right panel): Unedited western blot

♀

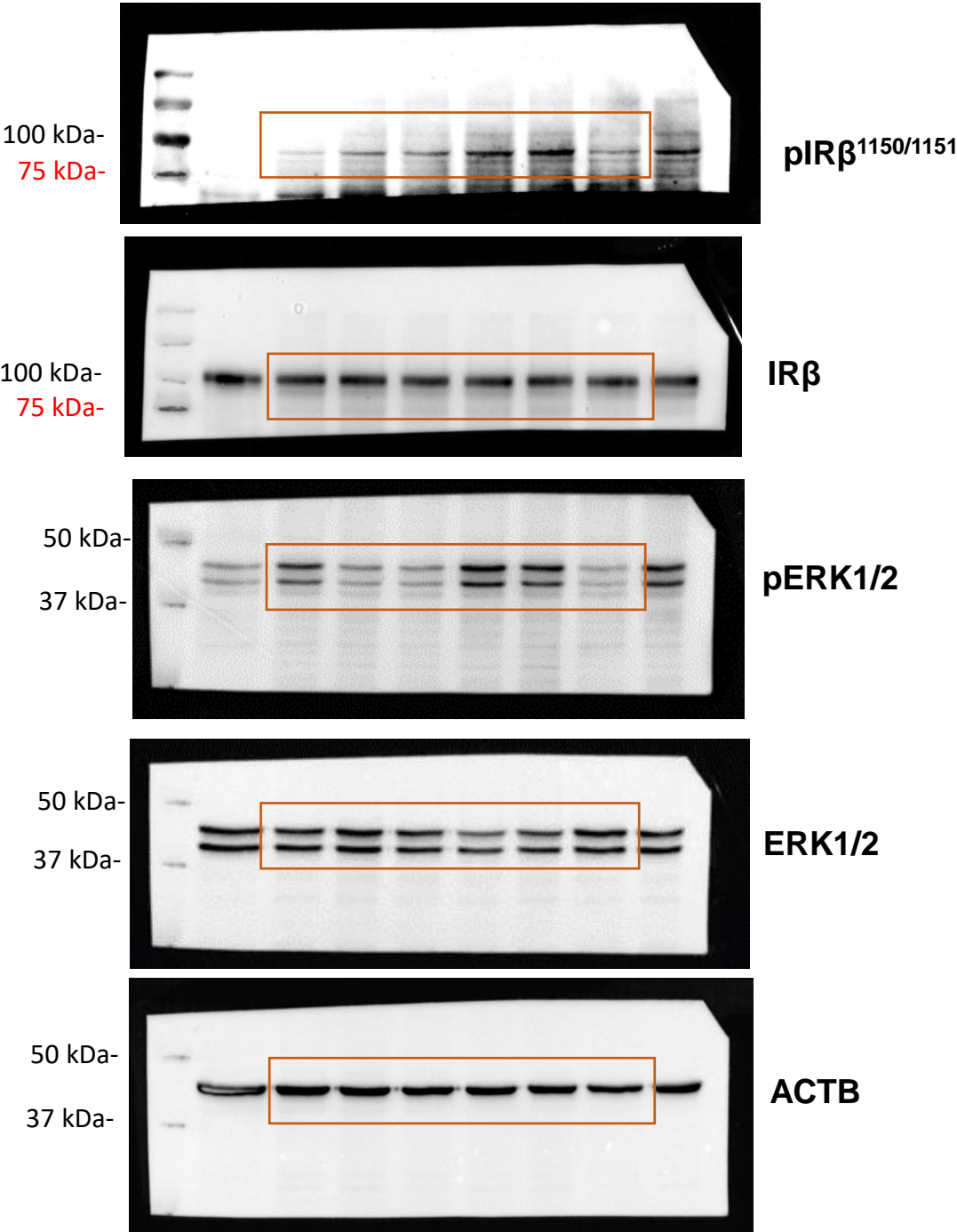

Supplement: Unedited blot and gel images [file jciinsight-11-203211-s282.pdf]
